# Health system use and experience among people with poor mental health: A cross-sectional analysis of the People’s Voice Survey in 18 countries

**DOI:** 10.1371/journal.pmed.1004745

**Published:** 2026-05-05

**Authors:** Margaret E. Kruk, Neena R. Kapoor, Catherine Arsenault, Susanne Carai, Federico M. Daray, Svetlana V. Doubova, Catherine K. Ettman, Patricia J. Garcia, Theodros Getachew, Ezequiel Garcia-Elorrio, Eric J. Lenze, Todd P. Lewis, Agustina Mazzoni, Jesus Medina-Ranilla, Sailesh Mohan, Inbarani Naidoo, Juhwan Oh, Emelda A. Okiro, Muhammad Pate, Marta B. Rondon, Rosanna Tarricone, Xiaohui Wang, Patricia Cavazos-Rehg

**Affiliations:** 1 Department of Medicine, School of Medicine, Washington University in St. Louis, St. Louis, Missouri, United States of America; 2 Department of Global Health and Population, Harvard T.H. Chan School of Public Health, Boston, Massachusetts, United States of America; 3 Department of Global Health, Milken Institute School of Public Health, The George Washington University, Washington, District of Columbia, United States of America; 4 World Health Organization Regional Office for Europe, Athens, Greece; 5 Instituto de Farmacología, Facultad de Medicina, Universidad de Buenos Aires, Buenos Aires, Argentina; 6 Centro de Educación Médica e Investigaciones Clínicas Norberto Quirno (CEMIC), National Scientific and Technical Research Council, Argentina (CONICET), Buenos Aires, Argentina; 7 Epidemiology and Health Services Research Unit, Mexican Institute of Social Security, Mexico City, Mexico; 8 Department of Health Policy and Management, Johns Hopkins Bloomberg School of Public Health, Baltimore, Maryland, United States of America; 9 School of Public Health, Cayetano Heredia University, Lima, Peru; 10 Health System Research Directorate, Ethiopia Public Health Institute, Addis Ababa, Ethiopia; 11 Institute for Clinical Effectiveness and Health Policy, Buenos Aires, Argentina; 12 Department of Psychiatry, School of Medicine, Washington University in St. Louis, St. Louis, Missouri, United States of America; 13 Centre for Chronic Disease Control, New Delhi, India; 14 Public Health Societies and Belonging, Human Sciences Research Council, KwaZulu-Natal, South Africa; 15 Seoul National University College of Medicine, Seoul, Republic of Korea; 16 Population and Health Impact Surveillance Group, Kenya Medical Research Institute-Wellcome Trust Research Programme, Nairobi, Kenya; 17 Nuffield Department of Medicine, Centre for Tropical Medicine and Global Health, University of Oxford, Oxford, United Kingdom; 18 Ministry of Health and Social Welfare of Nigeria, Abuja, Nigeria; 19 Instituto Nacional Materno Perinatal, Lima, Peru; 20 Department of Social and Political Sciences, Bocconi University, Milan, Italy; 21 Department of Health Management and Policy, School of Public Health, Lanzhou University, Lanzhou, China; Duke University, UNITED STATES OF AMERICA

## Abstract

**Background:**

Across the globe, rates of depression and anxiety have risen substantially since the COVID pandemic. Consequently, poor mental health is now a top health policy priority in many countries and more people than ever are seeking treatment. While the segment of people with poor mental health is large and growing, there is a dearth of data about their demographics and health needs and their use of and experience in the health system. Health systems require this information to effectively organize and provide services.

**Methods and findings:**

We investigated population prevalence of fair or poor mental health and compared health system experience and quality of care among adults with poor versus good mental health in 18 high-, middle-, and low-income countries using data from the People’s Voice Survey (*n* = 32,419). Data were collected in 2022 and 2023 through a combination of nationally representative telephone, online, and in-person surveys. Prevalence of self-reported poor mental health ranged from 4.7% in Nigeria to 39.6% in China and was unrelated to national income per capita. More women than men reported poor mental health in most countries. Across all countries, people with poor mental health had worse self-rated overall health and more chronic illness. Between 0.9% (Lao PDR) and 52.4% (UK) of those with poor mental health had received mental healthcare in the past year. People with poor mental health reported lower patient activation, worse care quality, and lower confidence in the health system. A study limitation is that results are based on self-reported mental health rather than clinical diagnoses.

**Conclusions:**

People with poor mental health have markedly different health profiles and health system experience. These findings should prompt health systems to re-assess their services to better serve this growing patient group. Comparison of user experience and quality over time and across countries with similar health systems may assist in benchmarking performance.

## Author summary

**Why was this study done?:**

- Rates of poor mental health have risen in many countries since the COVID-19 pandemic, but little comparable data exist on how people with poor mental health use and experience health systems across countries.
- People with poor mental health often have other health conditions and may face barriers to care, but global evidence on their broader health system experiences is limited.
- Health systems need population-level information to plan services and respond effectively to the growing population of patients with mental health needs.

**What did the researchers do and find?:**

- We analyzed nationally representative survey data from 32,419 adults in 18 countries collected in 2022–2023 through the People’s Voice Survey.
- The prevalence of self-reported poor or fair mental health ranged from 4.7% in Nigeria to 39.6% in China, and was not related to national income.
- Across countries, people reporting poor mental health were more likely to report chronic illness (41.8% versus 23.8%), worse overall health (69.6% versus 19.9%), lower patient activation, lower care quality, more unmet need, and lower confidence in being able to obtain and afford good care.

**What do these findings mean?:**

- People reporting poor mental health have distinct health profiles and more negative health system experiences, suggesting that health systems should strengthen integration of mental health into primary and first-contact care.
- Large differences across countries in mental healthcare use and confidence indicate opportunities for benchmarking and system improvement. While our survey was designed to permit comparability, inter-country differences in education, health system experience, and social and political context preclude direct comparison of all items.
- Our findings are based on self-reported mental health rather than clinical diagnoses, and because the study design is cross-sectional, the results show associations but cannot determine cause and effect.

## Abstract 3

In a survey study using the People's Voice Survey, Margaret Kruk and colleagues examine how people with poor mental health experience care and confidence in the health system across 18 countries.

## Introduction

Rates of poor mental, driven in large part by common mental disorders, such as depression and anxiety, have risen substantially in many countries since the COVID-19 pandemic [1–3]. As a result, populations have sought more care and some health systems are struggling to meet the increased demand [4,5]. Mood and anxiety disorders are priority conditions for health systems given that they are prevalent, responsible for a large burden of disease, and amenable to healthcare (e.g., psychotherapy, pharmacotherapy). Yet while the number of people living with poor mental health is growing globally, there is a lack of large-scale, comparable, post-pandemic data about their access to treatment, and use of and experience with health systems. For example, the World Mental Health Survey, a definitive source of information about the global mental health burden and care seeking, last collected data in 2019 [6,7].

Understanding the profile of people with poor mental health is vital for health systems planning, but population-level data are scarce in most countries. Studies from high-income countries suggest that mental health disorders are often co-morbid with common chronic diseases such as diabetes and cardiovascular disease [8,9]. Information on rates of comorbidity is important in particular for primary care, the entry point to the health system (including for mental healthcare) in most countries. The age and gender distribution of people with mental health conditions varies extensively across countries [10,11]. Patient activation, a measure of people’s knowledge and confidence in managing their own health, is associated with better health outcomes in depression and other conditions, but this is rarely measured in global surveys [12,13].

Health systems must also track their performance in addressing the mental health crisis. Rates of receipt of any depression treatment vary widely but have consistently been found to be very low in lower-income countries: only 15% of people with serious mental health needs reported receiving treatment in a landmark 2004 study [6]. Beyond access is the effectiveness of treatment: a recent re-analysis of the World Mental Health Surveys found that only 6.9% of people with common mental disorders received minimally effective treatment—that is, treatment that met basic clinical thresholds for pharmacotherapy or counseling [7].

Given different age distribution, underlying health, and stigma around mental disorders, people with poor mental health are likely to use general health services differently than those in good mental health and may have different experiences in their care. Connection to care, utilization, and quality ratings may be influenced by one’s mental health status [14,15]. Finally, confidence in the health system, a set of measures of health security and endorsement, which influence appropriate care seeking and which provide feedback essential for healthcare accountability may be influenced by mental health status [16].

In this paper, we use nationally representative data from the People’s Voice Survey in 18 countries to describe the characteristics of people with poor self-rated mental health and their use of healthcare post the COVID-19 pandemic. We explore their experience with care and confidence in the health system in their context, adjusting for individual and national factors. Taken together, these data provide a relatively comprehensive profile of a population in need that can inform how health systems organize care to meet the needs of people with poor mental health.

## Methods

### Ethics

The People’s Voice Survey was reviewed in accordance with local regulations in each participating country. The Harvard University Institutional Review Board deemed this research exempt, and local ethics approval was obtained where required. Written consent was not obtained because participation was anonymous and carried minimal risk. Respondents were informed about the study and participated voluntarily.

### Reporting guideline

This study is reported as per the *Consensus-Based Checklist for Reporting of Survey Studies (CROSS)* guideline (Table G in S1 Appendix).

### Study population

The research detailed in this paper was carried out by the Quality Evidence for Health System Transformation (QuEST) Network, an international research consortium focusing on high-quality health systems. This population-representative survey included 32,419 adult respondents across 18 countries. These countries included Ethiopia (excluding Tigray region), Kenya, Nigeria, South Africa, Peru, Colombia, Mexico, Uruguay, Argentina (province of Mendoza), Lao PDR, India, China, the Republic of Korea, Romania, Greece, Italy, the United Kingdom, and the United States. The sample of respondents per country ranged from 1,001 in Italy to 2,779 in Ethiopia. Respondents recruited through online panels were compensated in accordance with the terms of their panel enrollment. Telephone and in-person respondents did not receive compensation for survey participation.

### Data source

The data were collected using the People’s Voice Survey, a novel tool designed to measure individuals’ experiences and evaluations of health system performance. The survey captures information on demographics, health status, utilization of care, system competence, care experience, care quality, and confidence in the health system. The development and validation of this survey instrument have been previously documented [17]. We customized and translated the standard questionnaire to suit the context of each health system and assessed the country-specific questionnaires for comprehension through cognitive interviews and/or pilot tests.

QuEST Network-affiliated research teams contracted survey research firms Ipsos and SSRS to administer most of the surveys from May 2022 to April 2023 (see Table A in S1 Appendix). In Korea, Kstat conducted data collection. We gathered responses from population-representative samples of adults (aged 18 years and older) in each country. In most countries, surveys were conducted via computer-assisted telephone interviewing with a live interviewer. Respondents were selected through random digit dialing or known-list sampling. In Ethiopia and Kenya, where mobile phone penetration was below 80%, we supplemented telephone interviewing with face-to-face interviews. In the Republic of Korea, the United Kingdom, and the USA, we used nationally representative probability-based panels.

Gross domestic product (GDP) per capita data in each country were obtained from the World Bank, in purchasing power parities (PPPs), extracted from the World Bank in April 2025 [18]. PPPs adjust for price differences across countries to allow for more accurate comparisons of economic output and living standards.

### Indicators

The study utilized a range of standardized measures to assess mental health status, health status, healthcare utilization, quality of care, and health system confidence across participating countries.

#### Mental health assessment.

The primary outcome measure was a validated single-item self-reported mental health measure to assess the mental well-being of participants. The survey question asked respondents to rate their mental health, including their mood and ability to think clearly, as either “poor,” “fair,” “good,” “very good,” or “excellent.” For the purposes of this paper, responses of “poor” or “fair” were classified as poor mental health, while “good,” “very good,” and “excellent” were classified as good mental health, consistent with prior surveillance and research studies, such as the Behavioral Risk Factor Surveillance System (BRFSS) [19]. This measure has been shown to be strongly associated with diagnosis of concurrent and future major depressive disorder and use of antidepressants [20–24].

#### Health status and patient activation.

Self-rated overall health was assessed using a similar five-point scale, with responses categorized as either poor/fair or good/very good/excellent. Chronic illness was measured by self-report and has been shown to be associated with clinical presence of long-term health conditions [25]. Patient activation was defined as respondents reporting being “somewhat” or “very” confident they could bring up concerns to their healthcare provider and were “somewhat” or “very” confident they were responsible for managing their own health.

#### Healthcare utilization and access.

Several measures captured healthcare utilization patterns. Mental healthcare receipt was defined by self-reported care for mental health in the 12 months preceding the survey. Healthcare access was assessed through having a usual source of care (a regular healthcare provider or facility typically visited for medical care) and experiences of unmet need for care (needing healthcare services but not receiving them in the past 12 months). To illustrate differences in healthcare experiences between respondents with good (blue dots) versus poor mental health (orange dots), we utilized dumbbell plots for selected outcomes.

#### Quality of care.

Quality of care was measured through multiple indicators: respondents’ ratings of their last healthcare visit quality (categorized as “very good/excellent” versus “good/poor/fair”), experiences of unfair treatment or discrimination by healthcare providers, and assessment of their country’s mental health services quality (rated as “very good/excellent” versus “good/poor/fair”).

#### Health system confidence.

Confidence in health systems was evaluated through multiple measures: confidence in getting and affording good care (defined as responding “somewhat” or “very” confident to both getting good quality care and affording care), belief that the health system is getting better, and perception that the health system needs only minor changes rather than major reforms. We also assessed respondents’ ratings of government management of the COVID-19 pandemic on a scale from poor to excellent, as this factor could influence broader health system perceptions.

#### Sociodemographic factors.

Several sociodemographic factors were collected to analyze disparities and adjust for potential confounders. Age was categorized into three groups (18–29, 30–49, and 50+ years) to examine generational differences in health outcomes. Gender was self-reported, with respondents identifying as female, male, or another gender. Few respondents across all countries (*N* = 23) reported identifying as another gender, so for analytical purposes and consistent with other studies, these respondents were grouped females [26,27]. Education level was measured using country-specific categories standardized into low, medium, and high educational attainment for cross-country comparisons. Household income was assessed relative to national distributions and categorized into quintiles. Geographic location was captured as urban or rural residence based on national definitions. Insurance status recorded whether respondents had any form of health insurance coverage, including public, private, or mixed schemes.

All indicators were designed to facilitate cross-country comparisons while maintaining contextual validity across diverse healthcare systems and cultural settings.

### Statistical analysis

Post-stratification weights based on external population statistics were used to adjust the sample on variables including age, gender, region, and education, to reduce sampling biases (Table A in S1 Appendix). Weights were constructed using an iterative proportional fitting (raking) approach. For comparing proportions across groups, we calculated 95% confidence intervals (CIs) accounting for survey design.

Countries in the study were grouped by income using the World Bank country income groups (lower-middle-income, upper-middle-income, and high-income) to facilitate comparison within similar economies [28]. Accordingly, Nigeria, Kenya, Lao PDR, and India were treated as lower‑middle‑income economies; South Africa, Peru, Colombia, China, Mexico, and Argentina as upper‑middle‑income economies; and Uruguay, Greece, Romania, the Republic of Korea, Italy, the United Kingdom, and the United States as high‑income economies. Ethiopia was listed as unclassified in the World Bank’s FY26 income classification; however, it was previously classified as a low-income economy and thus was grouped with the other lower-middle-income countries for analytic purposes. For visualizing relationships between national wealth and mental health indicators, we plotted variables against GDP per capita in PPPs.

To analyze associations between mental health status and selected outcomes (health system confidence and mental healthcare uptake), we conducted logistic regression analyses. Models were adjusted for gender, age, education, income, patient activation, urban/rural residence, insurance status, presence of chronic illness, quality of usual source of care, and perceived government management of the COVID-19 pandemic, as these variables have been shown to influence healthcare ratings and use [16,29]. Statistical significance was denoted at *p* < 0.05, *p* < 0.01, and *p* < 0.001 levels.

All analyses were conducted using Stata version 18.0 (StataCorp, College Station, USA).

## Results

### Prevalence and distribution of poor or fair mental health

**Fig 1:**
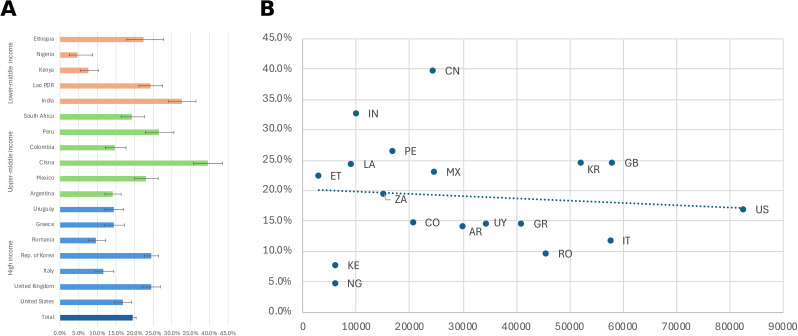
Prevalence of fair or poor mental health across 18 countries. **(A)** Weighted percent of respondents who reported “poor” or “fair” on a five-point scale in response to the question: “Rate your mental health, including your mood and ability to think clearly.” Error bars represent 95% CI, calculated accounting for survey design. Countries are grouped by income classification (blue = high income, green = middle income, orange = lower income). **(B)** GDP per capita is shown in purchasing power parities (PPPs), which adjust for price differences across countries to allow for more accurate comparisons of economic output and living standards. The dotted line represents the linear trend across countries. Country codes: AR, Argentina; CN, China; CO, Colombia; ET, Ethiopia; GB, United Kingdom; GR, Greece; IN, India; IT, Italy; KE, Kenya; LA, Lao PDR; MX, Mexico; NG, Nigeria; PE, Peru; RO, Romania; US, United States; UY, Uruguay; ZA, South Africa. See Table B in S1 Appendix for exact percentages and GDP (PPP).

The prevalence of poor or fair self-reported mental health (labeled here as “poor mental health” for parsimony) varied substantially across the 18 countries in the study (Fig 1A). Overall, nearly one in five respondents (19.5%) reported poor or fair mental health, with rates ranging from 4.7% in Nigeria to 39.6% in China. Other countries with high prevalence included India (32.7%) and Peru (26.5%). Among high-income countries, both the UK and the Republic of Korea reported prevalence exceeding 20%. We found no clear relationship between country wealth (GDP) and the prevalence of poor mental health (Fig 1B), suggesting that factors beyond economic development influence population mental health status.

**Fig 2:**
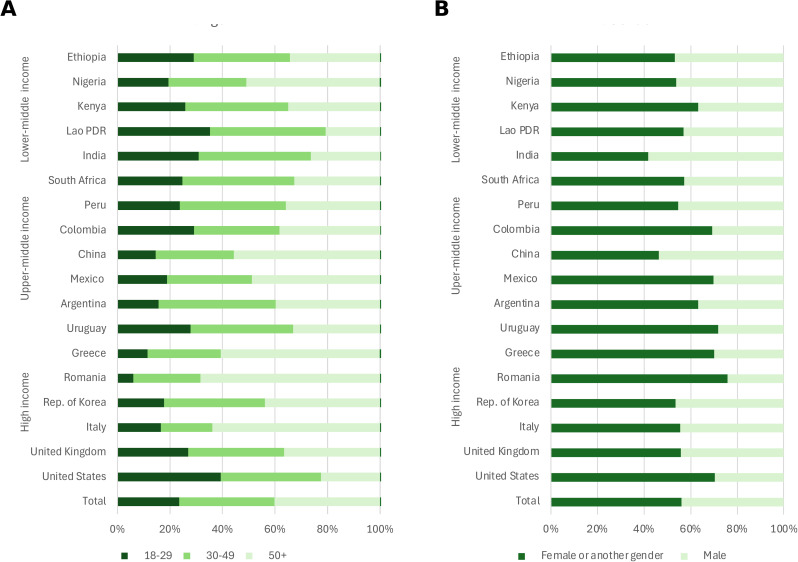
Age and gender distribution of people with poor mental health. **(A)** Distribution of respondents reporting “poor” or “fair” mental health by age group (18–29, 30–49, and 50+ years). **(B)** Distribution by gender; dark green bars represent female or another gender respondents and light green bars represent male respondents. All percentages are adjusted using survey weights to ensure national representativeness. Countries are grouped by income classification (high, middle, and lower income). Total bars represent the population-weighted average across all surveyed countries. See Table C in S1 Appendix for exact percentages.

The demographic distribution of poor mental health varied across countries (Fig 2A). In terms of age, several patterns emerged. In many countries, including Italy, Greece, Romania, China, Mexico, and Nigeria, more people with poor mental health were older adults (50+ years). In contrast, in the United States, India, and Lao PDR, poor mental health was more evenly distributed across age groups or skewed toward younger (18–29) or middle-aged (30–49) respondents. Gender differences were apparent, with female representing a larger proportion of those with poor mental health in all countries except China and India (Fig 2B). The largest differences were observed in middle-income countries, including several Latin American nations, as well as in Greece, Romania, and the United States.

### Health status and patient activation

**Fig 3:**
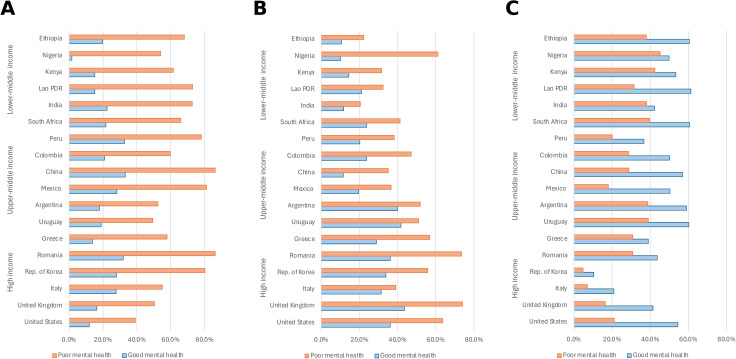
Health status and patient activation by mental health status. **(A)** Percentage reporting good, very good, or excellent self-rated health. **(B)** Percentage reporting a self-reported chronic illness. **(C)** Percentage with high patient activation, defined as respondents who reported being “somewhat” or “very” confident they could bring up concerns to their healthcare provider and were “somewhat” or “very” confident they were responsible for managing their own health. In all panels, orange bars represent respondents with poor/fair mental health and blue bars represent those with good/very good/excellent mental health. Mental health categorization is based on respondents’ self-rating on a five-point scale. Countries are grouped by income classification. All percentages are adjusted using survey weights. Asterisks indicate statistical significance (*p < 0.05, **p < 0.01, ***p < 0.001). See Table C in S1 Appendix for exact percentages.

People with poor mental health reported substantially worse overall health compared to those with good mental health across all countries (Fig 3A). While 19.9% of respondents with good mental health reported their general health as poor or fair, 69.6% of those with poor mental health did so. This large difference in self-reported health status was consistent across countries at all income levels, highlighting the association between self-reported mental and physical health.

Chronic illness was significantly more prevalent among respondents with poor mental health (Fig 3B). Across all countries, 41.8% of those with poor mental health reported having a chronic condition, compared to 23.8% of those with good mental health. These differences were consistent across income classifications, with the largest disparities observed in high-income countries.

Patient activation, defined as confidence in managing one’s health and communicating concerns with providers, was substantially lower among those with poor mental health (Fig 3C). While 48.1% of respondents with good mental health demonstrated high patient activation, only 28.7% of those with poor mental health did so. This pattern was consistent across all income groups and particularly pronounced in lower-income countries.

### Mental health care receipt and access to care

**Fig 4:**
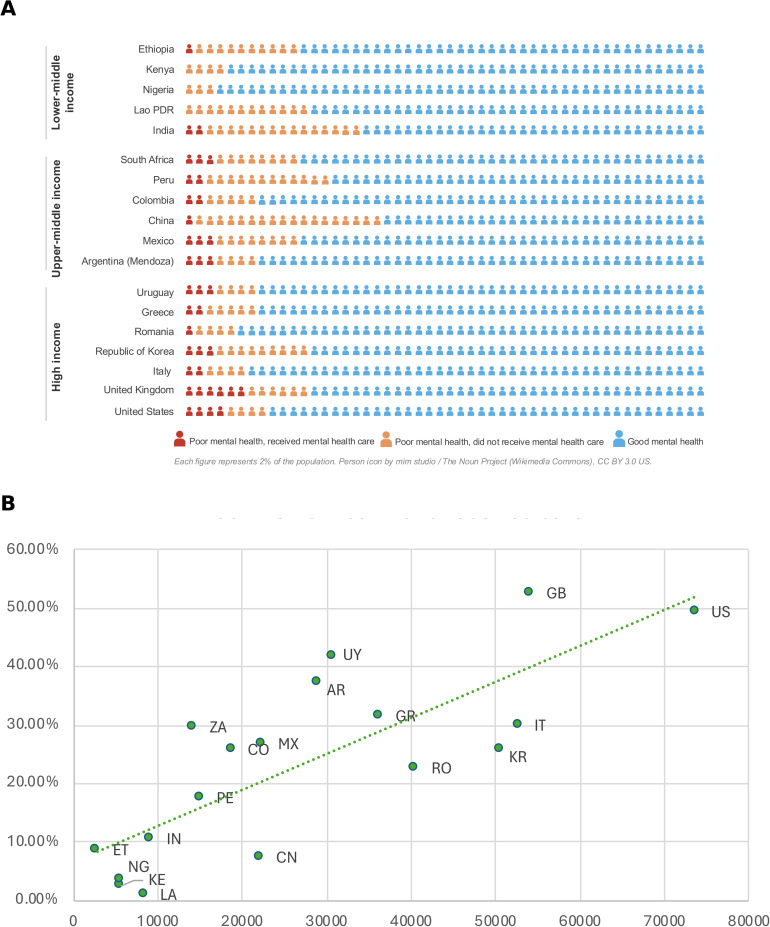
Mental healthcare receipt among people with poor mental health. **(A)** Infographic displaying the weighted distribution of mental health status and care receipt. Each figure icon represents 2% of the population. Dark orange = respondents with poor/fair mental health who received mental healthcare in the past 12 months; light orange = those with poor/fair mental health who did not receive care; blue = those with good/very good/excellent mental health. **(B)** Percentage of respondents with poor/fair mental health who received mental healthcare, plotted against GDP per capita in purchasing power parities (PPPs). The dotted green line represents the linear trend. Poor mental health is defined as respondents who reported “poor” or “fair” on a five-point self-rating scale. All percentages are adjusted using survey weights. Country codes: AR, Argentina; CN, China; CO, Colombia; ET, Ethiopia; GB, United Kingdom; GR, Greece; IN, India; IT, Italy; KE, Kenya; KR, Republic of Korea; LA, Lao PDR; MX, Mexico; NG, Nigeria; PE, Peru; RO, Romania; US, United States; UY, Uruguay; ZA, South Africa. See Table B in S1 Appendix for exact percentages and GDP (PPP).

The proportion of people with poor mental health who received mental healthcare in the past 12 months varied dramatically across countries (Fig 4A and 4B). Mental healthcare receipt was strongly associated with national income levels; less than 4% of respondents with poor mental health in Kenya (2.6%), Nigeria (3.4%), and Lao PDR (0.9%) reported receiving mental healthcare, compared to 52.4% in the United Kingdom and 49.3% in the United States.

**Fig 5:**
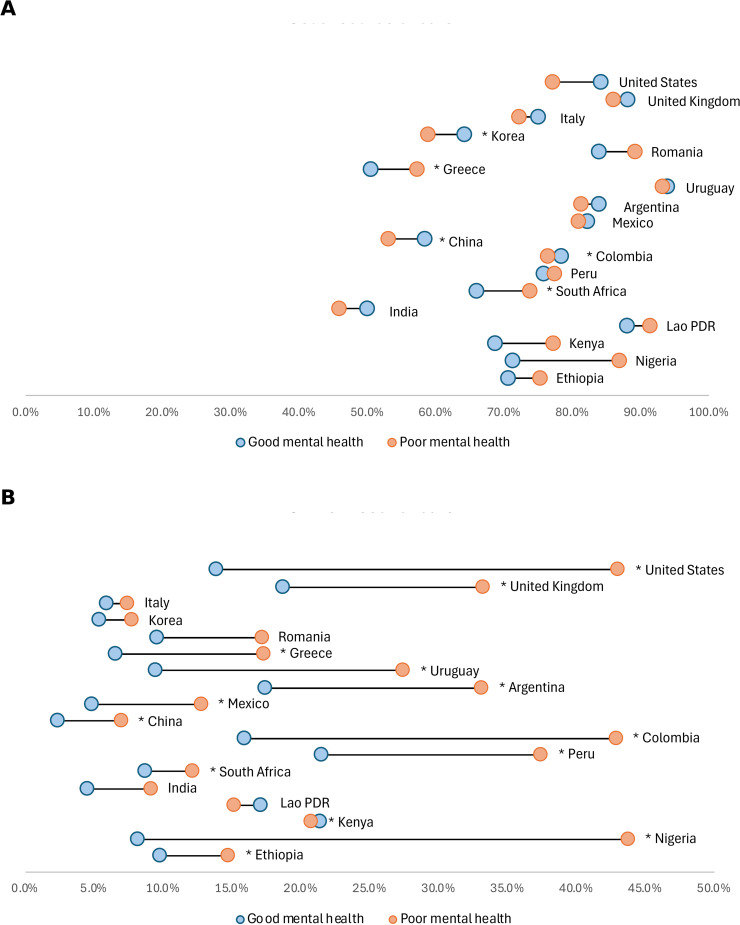
Access to healthcare by mental health status. **(A)** Percentage with a usual source of care (a regular healthcare provider or facility). **(B)** Percentage reporting unmet need for healthcare in the past 12 months. In both panels, connected dots compare those with good mental health (blue) and poor mental health (orange). Poor mental health is defined as respondents who reported “poor” or “fair” on a five-point self-rating scale, while good mental health includes those reporting “good,” “very good,” or “excellent.” All percentages are adjusted using survey weights. Asterisks indicate statistical significance based on survey-weighted logistic regression (*p < 0.05). See Table C in S1 Appendix for exact percentages.

Access to healthcare also differed by mental health status. In several countries, including the United States, the United Kingdom, Italy, the Republic of Korea, China, India, Argentina, Uruguay, and Mexico, respondents with poor mental health were less likely to have a usual source of care compared to those with good mental health (Fig 5A). Similarly, people with poor mental health were significantly more likely to report unmet healthcare needs in the past 12 months (Fig 5B).

In a pooled analysis of all data, controlling for a range of individual covariates and including country fixed effects, we found that receipt of care was positively associated with female gender (OR 1.51, 95% CI [1.28, 1.78]; *p* < 0.001), having public insurance (versus none) (OR 1.36, 95% CI [1.00, 1.84]; *p* = 0.048), having chronic illness (OR 2.31, 95% CI [1.95, 2.74]; *p* < 0.001), having a usual source of care that was poor, fair, or good (versus none) (OR 1.26, 95% CI [1.03,1.54]; *p* = 0.025) and having a usual source of care that was very good or excellent (versus none) (OR 1.53, 95% CI [1.22, 1.92]; *p* < 0.001). Age 50 or greater was negatively associated with receipt of mental healthcare (OR 0.69, 95% CI [0.55, 0.87]; *p* = 0.002). (Table E in S1 Appendix)

### Quality of care and health system confidence

**Fig 6:**
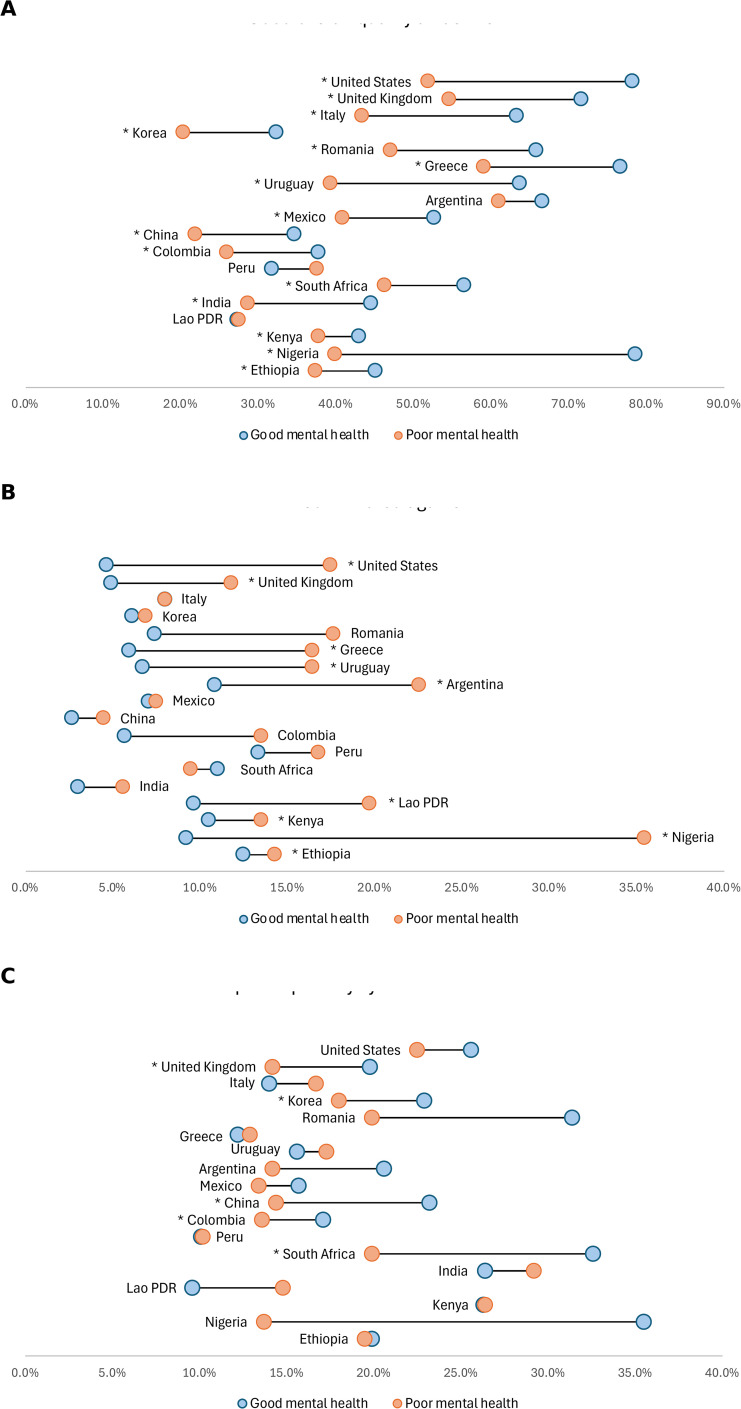
Quality of care by mental health status. **(A)** Percentage rating the overall quality of their last healthcare visit as “very good” or “excellent.” **(B)** Percentage reporting unfair treatment or discrimination by a health provider. **(C)** Percentage rating the quality of their country’s mental health services as “very good” or “excellent.” In all panels, connected dots compare those with good mental health (blue) and poor mental health (orange). Poor mental health is defined as respondents who reported “poor” or “fair” on a five-point self-rating scale, while good mental health includes those reporting “good,” “very good,” or “excellent.” All percentages are adjusted using survey weights. Asterisks indicate statistical significance (*p < 0.05, **p < 0.01, ***p < 0.001). See Table C in S1 Appendix for exact percentages.

Respondents with poor mental health consistently rated their healthcare experiences less favorably than those with good mental health. Across most countries, they were significantly less likely to rate their last healthcare visit as “very good” or “excellent” (Fig 6A). They also reported higher rates of unfair treatment or discrimination by healthcare providers (Fig 6B), with particularly large disparities observed in Nigeria, the United States, and Argentina. When rating the quality of mental health services in their country, respondents with poor mental health gave lower ratings than those with good mental health in most countries (Fig 6C).

**Fig 7:**
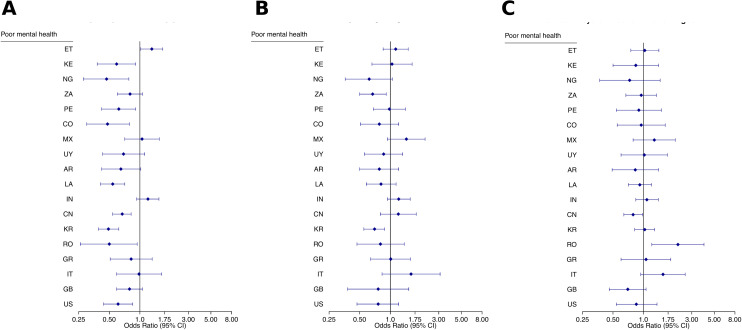
Confidence in the health system by mental health status, adjusted. Forest plots showing adjusted odds ratios (with 95% CI) among those with poor mental health compared to those with good mental health across 18 countries. **(A)** Confidence in getting and affording good care. **(B)** Belief that the health system is getting better. **(C)** Perception that the health system needs only minor changes. Poor mental health is defined as respondents reporting “poor” or “fair” mental health on a five-point self-rating scale. Models are adjusted for gender, age, education, income, patient activation, urban/rural residence, insurance status, presence of chronic illness, quality of usual source of care, and perceived government management of the COVID-19 pandemic. Country codes: AR, Argentina; CN, China; CO, Colombia; ET, Ethiopia; GB, United Kingdom; GR, Greece; IN, India; IT, Italy; KE, Kenya; KR, Republic of Korea; LA, Lao PDR; MX, Mexico; NG, Nigeria; PE, Peru; RO, Romania; US, United States; UY, Uruguay; ZA, South Africa. See Table D in S1 Appendix for exact adjusted odds ratios and 95% CI.

After adjusting for demographic and health system factors, poor mental health was associated with significantly lower confidence in getting and affording good healthcare in most countries (Fig 7A). In 9 of 18 countries, respondents with poor mental health had significantly reduced odds of expressing confidence that they could get and afford good care if seriously ill. These associations were particularly strong in Colombia (OR 0.48, 95% CI [0.30, 0.79]; *p* = 0.004), Nigeria (OR 0.47, 95% CI [0.28, 0.78]; *p* = 0.004) and Korea (OR 0.49, 95% CI [0.39, 0.62]; *p* < 0.001). In contrast, beliefs about health system improvement (Fig 7B) and perceptions that the health system needs only minor changes (Fig 7C) showed more varied patterns, with fewer consistent associations with mental health status.

**Fig 8:**
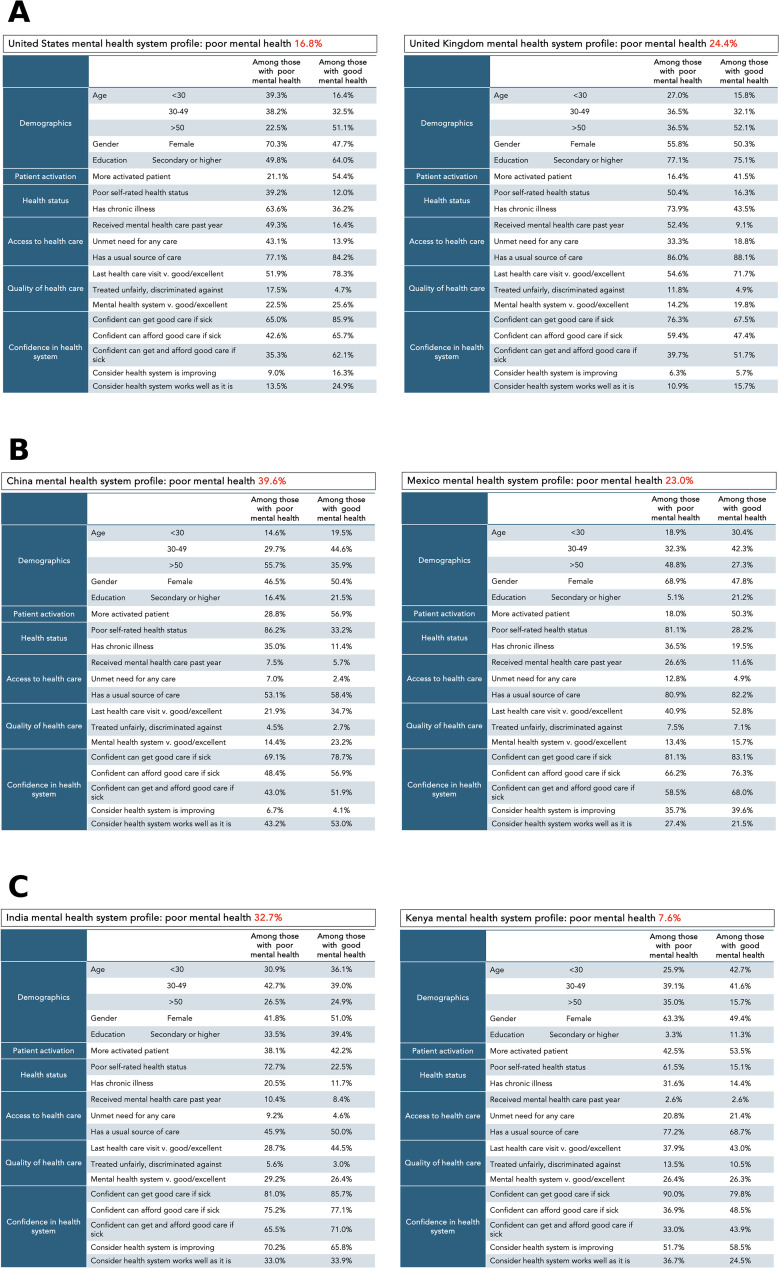
Mental health system profiles for selected countries. Country-level profiles comparing key indicators between respondents with poor and good mental health across six domains: demographics, patient activation, health status, access to healthcare, quality of healthcare, and confidence in the health system. **(A)** High-income countries: United States and United Kingdom. **(B)** Upper-middle-income countries: China and Colombia. **(C)** Lower-middle-income countries: India and Ethiopia. Poor mental health is defined as respondents who reported “poor” or “fair” on a five-point self-rating scale. All percentages are adjusted using survey weights.

Fig 8 presents comprehensive mental health system profiles for selected countries across different income classifications, highlighting the interconnections between mental health status, comorbidities, healthcare access, and system experiences in different contexts. These profiles reveal distinct patterns of strengths and challenges in addressing mental health needs across healthcare systems at different resource levels.

## Discussion

This study of 18 low-, middle-, and high-income countries yielded several key findings. First, there are substantial differences among countries in the proportion of people who reported poor or fair mental health (labeled “poor mental health” in this paper) and in the age, gender, and health needs of those individuals. Second, comorbidity rates are overall high, suggesting an essential role for primary care. Third, there are major differences in use of mental healthcare; these are strongly associated with national income. Fourth, while countries provide a range of care quality, people with poor mental health almost universally report worse quality and user experience in accessing general health services and tend to have lower confidence in the health system.

We found a substantial prevalence of self-reported poor or fair mental health—a measure strongly correlated with depression—two to three years post the start of the COVID-19 pandemic. Poor mental health is not the province of rich countries—we found that prevalence was unrelated to national income, suggesting that factors other than economic resources or living standards are important drivers. Additionally, report of mental health conditions is shaped by cultural norms and societal context—particularly how individuals are conditioned to perceive, articulate, or even acknowledge mental health issues [30–32].

China had the highest proportion reporting poor mental health at nearly 40%, followed by India at 33%. Among high-income countries, the United Kingdom and Korea had prevalence in excess of 20%. While question wording and the time horizon for measuring prevalence of mental health conditions (e.g., today, two-week, past year) differ across studies, our estimates broadly comport with the few available measures in the same time frame. We find 16.8% point prevalence in the US in 2023 whereas the National Institute of Mental Health reports 23.1% past year prevalence of any mental illness in 2022 [33]. Li and colleagues found 23% prevalence of presumptive depression in China in 2023 compared to our figure of 39.6% in poor/fair mental health [34]. Their measure and cutoff for depression threshold was higher (PHQ-9 >10), which may account for their lower prevalence.

Women outnumbered men among people with poor mental health in all countries, except China. The largest female-to-male differences were in middle-income countries, including countries in Latin America, and in Greece, Romania, and the United States. Global studies have similarly found that women are more likely than men to suffer from mental illness, attributing this to genetics and social inequalities [35]. In terms of age, we found broadly equal distribution of age groups (younger <30, middle-aged 30–49, and older people >50) among people with poor mental health in most countries. However, ages in Nigeria, China, Greece, Romania, and Italy, skewed older, with people over 50 years comprising half or more of cases. While global data show that younger people have a higher risk of anxiety and depression, the large and growing population of older people worldwide means that in absolute terms, older people comprise a large group in need [36]. This points to the need for health systems to pursue a range of strategies for detecting and treating people with depression in diverse age groups to meet their needs and maximize treatment effectiveness [37,38].

We found striking differences in poor self-rated health and rates of chronic illness among people with poor mental health compared to those with good mental health. Across the countries in this analysis people with poor mental health were more than nine times as likely as those with good mental health to report poor overall health and more than twice as likely to have an ongoing or chronic condition. Our findings of an association between poor mental health and physical illness comport with global data showing high rates of concurrent illness among people with depression and anxiety [9,39]. Scott and colleagues found that most common mental disorders such as mood, anxiety, substance use, and impulse control disorders were significantly associated with seven to 10 common chronic physical conditions in 17 countries [39]. This relationship between depression and chronic conditions is likely bidirectional and underlines the need for health systems to screen people with common mental disorders, including young people, for chronic conditions and vice versa [39,40]. Study respondents reporting poor mental health were 60% less likely to be activated, that is they are less likely to take initiative on their health. This has important implications for treatment success. Sacks and colleagues found that more activated patients had higher rates of remission—up to 2-fold for the most activated—likely through greater engagement in healthy behaviors [12].

Across the countries in our study, 20.0% of respondents with poor mental health on the day of the survey reported receiving any care for depression, anxiety, or another mental health disorder in the past 12 months. This is not a direct measure of treatment access for depression and anxiety as our outcome is not a diagnosis of these disorders. Depressive symptoms may wax and wane in the general population and can resolve without clinical treatment; thus a portion of our respondents may not require mental healthcare. However, Vigo and colleagues found that people with more severe mental disorders are not more likely to obtain treatment than those with less serious disorders [6]. They and others have found that perceived need for mental healthcare is more predictive of treatment use than severity [41].

Our country-level findings on the use of mental healthcare are consistent with other research. We found that in China 7.5% of people with poor mental health received mental healthcare; Lu and colleagues reported fewer than 10% of respondents with major depression reported receiving any mental health treatment in 2021 [42]. As with other work, we found that mental health treatment was strongly associated with national income with less than 5% of people with poor mental health using care in Laos, Kenya, and Nigeria versus 49% in the US and 53% in the UK [6,43]. One likely major factor is the shortage of mental health professionals and overall low health system investment in mental healthcare, which is especially acute in lower-income countries [44–47]. Several individual factors also predicted mental care use. In pooled controlled analyses, women (OR 1.51), people with chronic disease (OR 2.31), people with public insurance versus no insurance (OR 1.36), as well as people with a usual source of care that they rated as very good or excellent (OR 1.53) were more likely to receive care (Table E in S1 Appendix). People over 50 were less likely to receive care (OR 0.69). These patterns are similar to other studies [7,43,48]. This suggests that repeated contact with health systems can promote uptake of needed mental healthcare.

In terms of overall health system use, people with poor mental health were slightly less likely to have a usual source of care, with statistically significant gaps in Korea (OR 0.8), China (OR 0.8), and Colombia (OR 0.7) (Table F in S1 Appendix). In Greece and South Africa people with poor mental health had a higher likelihood of having a usual source (OR 1.5 and OR 1.3, respectively). However, despite having similar access, more people with poor mental health than good mental health reported unmet need for healthcare in the past year: after controlling for a range of covariates, unmet need was more than 2-fold higher in 8 of 18 countries (Table F in S1 Appendix). This may be a combination of greater need for all medical services given higher prevalence of comorbidity, greater perception of health need, and a range of barriers to care, including difficulty navigating complex health systems and stigma [7,41].

Quality of care was rated more negatively among people with poor mental health in most countries. Overall, people reported lower ratings of care quality and a higher rate of unfair or discriminatory treatment by health providers compared to people with good mental health. Our quality measure was not restricted to mental healthcare, so reflects a broader assessment of the health system by this population. Our finding supports past research showing lower ratings for quality of care among people with depressive disorders or low affect [49–52]. This may be related to user factors (dissatisfaction with life in general) and provider factors (negative reaction to people with depression)—or likely a combination [53]. Regardless of the mechanism, poor perceived quality of general health services may deter care seeking for depression and adherence to treatment [43,54,55].

Our findings on confidence in the health system were mixed. Endorsement for current health systems and recent trajectory was overall low but comparable to respondents with good mental health [16]. However, health security (confidence that you can get and afford good care if very sick tomorrow) was substantially lower among people with poor mental health, even after controlling for demographic factors and country context [16,29]. There is little comparable work on poor mental health and health security, but there are well-demonstrated associations between poor overall health and chronic disease and lower confidence in the health system [16,56]. Low confidence in the health system may undermine the social compact needed to sustainably fund universal health coverage [16].

Our study has several limitations. Our outcome measure of poor mental health is not diagnostic of depression or anxiety, although it is correlated with both and has been validated for population estimates of mental health burden in past work [20–24]. While our survey items were designed to permit comparability, inter-country differences in education, health system experience, and social and political context preclude direct comparison of some items, such as health system ratings [16]. Awareness of mental health and levels of stigma vary across countries, potentially affecting ability and willingness to report poor self-rated mental health; however, the item we used employs ‘lay’ rather than clinical wording and has been extensively used in a variety of settings in past work. We used an abbreviated activation measure—given the strong association with poor mental health and treatment future research should explore this in more detail. We focus here primarily on individual country results and limit comparison to countries at similar economic levels. While in our pooled regression we found that having public health insurance is associated with uptake of mental healthcare, differences in insurance design, scope, and inclusion of mental health benefits, precludes any specific conclusions about the role of insurance in promoting care uptake. Although half of all mental health problems begin in adolescence and account for 15% of the disease burden in this age group—with notable increases during and after the COVID-19 pandemic—our study currently includes only adults. To address this limitation, the PVS tool has been adapted to capture the experiences of adolescents aged 15 years and older. Data collection is finalized, underway, or planned in Germany, Latvia, the Netherlands, Scotland, and Switzerland. We were unable to collect data in the Tigray region of Ethiopia due to ongoing conflict; this may underestimate Ethiopia’s prevalence of poor mental health given that populations exposed to conflict have greater rates of depression and anxiety [57]. Finally, our work is largely descriptive, a detailed examination of system-level drivers of mental healthcare access, use, and experience, is an important area for future research.

Our work also has a number of strengths. Ours is one of few studies to estimate poor mental health and healthcare experience across countries since the pandemic. Unlike other work focused on uptake of mental healthcare, we evaluate broader health system use and experience. Our sample sizes are large and nationally representative and our survey items were validated in past research and in extensive formative work [17].

These findings have a number of implications for health systems. First, countries need to understand the demographic, health, and health system profile of people with poor mental health. We created several sample health system profiles that aggregate key data about this population, comparing countries at similar economic levels. These can be used to inform health system planning and to benchmark countries with similar healthcare resources to guide system improvement. For example, more people with poor mental health report unmet need for care and lack of usual source of care in the US than in the UK, despite higher health expenditures in the former. And in both countries, the rate of chronic illness is nearly twice as high and the rate of activation half as high as for people with good mental health. Measuring and through therapy and education, targeting patient activation may be an important mechanism for improving or accelerating remission [58,59].

With regards to health system improvement, integrating diagnosis and, where resources are available, treatment of common mental disorders into primary care or other first-contact care should be a priority [60,61]. This is consistent with long-standing global guidance [62]. Given high rates of chronic illness, closer attention to detecting and treating chronic conditions for patients presenting with poor mental health is an important opportunity for global health improvement—one such model is collaborative care [63,64]. The broad distribution of ages points to the need for differentiated services, such as in-person, telehealth and app and internet care, school- and community-based care models to meet the needs and preferences of younger versus older patients [65–67]. Men used less mental healthcare in our study and thus may also need greater encouragement to access treatment. Men and women also have different treatment preferences with women being more receptive of psychotherapy and men pharmacotherapy [68–70].

Quality of care needs urgent attention given the lower perceived quality ratings of general and mental health services among people with poor mental health in our study. While some of the difference in quality ratings are due to reporting differences related to low affect and thus do not conclusively indicate inferior clinical care, it remains the case that people with poor mental health are more unhappy than others with their care. This has implications for individuals’ engagement in their own care and ultimately their health outcomes. Paying greater attention to responsiveness and quality of general medical services, including stronger integration of mental health into primary care, care coordination, and clinician empathy, will benefit this group and the entire patient population. Countries are increasingly promoting integration into primary care in their strategic plans, but how these plans function for health system users in practice is understudied [71]. For example, promoting rapid treatment after diagnosis and building a strong therapeutic compact with providers can be powerful drivers for improving remission rates of mental disorders [72,73]. Given the large societal and human costs of undertreated mental health disorders, investments in better care systems are likely to be cost-effective [74].

As health systems across countries at all income levels encounter increasing numbers of people with mental health disorders, actionable intelligence on their healthcare experiences and health needs can assist in updating care models to best serve this subgroup.

## Supporting information

S1 Appendix **Table A.** Survey methods for study countries. **Table B.** GDP per capita, self-reported mental health status, and care for mental health across 18 countries. **Table C.** Demographic factors, health status, healthcare utilization, and quality of care by mental health status across 18 countries. **Table D1.** Association between poor mental health status and confidence in the health system, adjusted. **Table D2.** Association between poor mental health status and confidence in the health system, unadjusted. **Fig A.** Confidence in the health system for people in poor mental health, unadjusted. **Table E.** Factors associated with receipt of mental healthcare among people with poor mental health. **Table F.** Association between poor mental health status and confidence in the health system, unadjusted. **Table G.** CROSS Checklist.
(DOCX)

## Abbreviations

BRFSS: Behavioral Risk Factor Surveillance System
CI: confidence interval
GDP: gross domestic product
PPPs: purchasing power parities
QuEST: Quality Evidence for Health System Transformation
