# Supplementary material for: Health system use and experience among people with poor mental health: A cross-sectional analysis of the People’s Voice Survey in 18 countries
Source: PLoS Med. 2026 May 5;23(5):e1004745. doi: 10.1371/journal.pmed.1004745 (PMC13143085; doi:10.1371/journal.pmed.1004745)
Supplement: S1 Appendix — Table A. Survey methods for study countries. Table B. GDP per capita, self-reported mental health status, and care for mental health across 18 countries. Table C. Demographic factors, health status, healthcare utilization, and quality of care by mental health status across 18 countries. Table D1. Association between poor mental health status and confidence in the health system, adjusted. Table D2. Association between poor mental health status and confidence in the health system, unadjusted. Fig A. Confidence in the health system for people in poor mental health, unadjusted. Table E. Factors associated with receipt of mental healthcare among people with poor mental health. Table F. Association between poor mental health status and confidence in the health system, unadjusted. Table G. CROSS Checklist. (DOCX) [file pmed.1004745.s001.docx]

**S1 Appendix**

**Table A Survey methods for study countries**

| **Country (wave)** | **Survey version** | **Average survey length by mode (minutes)** | **Fieldwork dates** | **Survey languages** | **Data collection partner** | **Response rate (%) *** | **Sample sizes** | **Sampling frame (mode)** | **Weighting variables** |
| --- | --- | --- | --- | --- | --- | --- | --- | --- | --- |
| Colombia (CO) | 1 | CATI: 33.58 | 7/7/22 – 8/23/22 | Spanish | Ipsos | 13% | 1,237 | RDD (CATI) | Age, gender, region, education |
| Ethiopia (ET) | 1 | CATI: 24.12; FTF: 24.11 | 8/16/22 –11/4/22 | Afan Oromo, Amharic, Tigrigna, Somali | Ipsos | 32% (CATI); 58% (FTF) | 2,445 (CATI); 334 (FTF) | Known-list sampling (CATI) and multi-stage clustered design (FTF) | Age, gender, region, education |
| India (IN) | 1 | CATI: 23.17 | 2/20/23 - 4/3/23 | English, Hindi, Marathi, Tamil, Telegu, Bengali, Assamese, Gujarati, Kannada | Ipsos | 8% | 2,004 | RDD (CATI) | Age, gender, region, education |
| Kenya (KE) | 1 | CATI: 19.64; FTF: 25.52 | 8/17/22 – 10/08/22 | Swahili, English | Ipsos | 35% (CATI); 84% (FTF) | 2,006 (CATI); 299 (FTF) | RDD (CATI) and multi-stage clustered design (FTF) | Age, gender, region, education |
| Peru (PE) | 1 | CATI: 24.69 | 7/05/22 – 9/15/22 | Spanish | Ipsos | 6% | 1,255 | RDD (CATI) | Age, gender, region (Lima and other), education |
| South Africa (ZA) | 1 | CATI: 26.61 | 10/20/22 - 1/20/23 | English, Afrikaans, isiXhosa, isiZulu, Sepedi, Sesotho, Setswana | Ipsos | 10% | 2,036 | RDD (CATI) | Age, gender, region, education |
| Uruguay (UY) | 1 | CATI: 18.68 | 7/12/22 – 9/19/22 | Spanish | Ipsos | 8% | 1,237 | RDD (CATI) | Age, gender, region (2 largest regions and other), education |
| Laos (LA) | 1 | CATI: 22.26 | 5/9/22 – 8/19/22 | Lao, Hmong, Khmou | In-house | 18% | 2,007 | RDD (CATI) | Age, gender, region, education, urban/rural |
| United States (US) | 1 | CAWI: 16.37; CATI: 27.63 | 12/14/22 - 1/23/23 | English, Spanish | SSRS | 2% | 50 (CATI); 1,450 (CAWI) | Online probability panel | Age, gender, race/ethnicity, education, census region, population density, civic engagement, internet use frequency, voter registration status, party ID, and religious affiliation |
| Mexico (MX) | 1 | CATI: 22.67 | 12/21/22 - 1/31/23 | Spanish | SSRS | 3% | 1,002 | RDD (CATI) | Age, gender, education |
| Italy (IT) | 1 | CATI: 22.71 | 12/16/22 - 1/20/23 | Italian | SSRS | 5% | 1,001 | RDD (CATI) | Age, gender, education |
| Republic of Korea (KR) | 1 | CAWI: 19.72 | 2/10/23 - 2/16/23 | Korean | Kstat | 5% | 2,000 | Online probability panel | Age, gender, region, education (through stratified sampling) |
| Argentina (AR) | 1 | CATI: N/A | 9/22/22 - 11/16/22 | Spanish | Ipsos | 4% | 1,190 | Known-list sampling | Age, insurance coverage, region |
| United Kingdom (GB) | 1 | CATI: 29.9; CAWI: 17.2 | 3/17/23 - 3/29/23 | English | SSRS | 4% | 92 (CATI); 1,585 (CAWI) | Online probability panel | Age, gender, and education |
| Greece (GR) | 1 | CATI: 18.03 | 5/17/23 - 7/16/23 | Greek | Ipsos | 18% | 2,010 | RDD (CATI) | Age, gender, region, education, urban/rural |
| China (CN) | 2 | CATI: 14.5 | 10/11/23 - 12/21/23 | Mandarin | Ipsos | 1% | 2,625 | RDD (CATI) | Age, gender, region, education |
| Nigeria (NG) | 1 | CATI: 28.76 | 6/1/2023 -7/5/2023 | Hausa, Igbo, Pidgin, Yoruba, English | Ipsos | 26% | 2,555 | RDD (CATI) | Age, gender, region |
| Romania (RO) | 1 | CATI: 26.2 | 05/06/2023 - 06/30/2023 | Romanian | Ipsos | 12% | 2001 | RDD (CATI) | Age, gender, region, education |

* Response rates for the PVS are calculated following standard practices in the survey literature, including approaches used by large surveys such as the Commonwealth Fund International Health Policy (IHP) survey. Specifically, we use the American Association for Public Opinion Research (AAPOR) Response Rate 3, which is defined as the number of completed interviews divided by the sum of completed interviews, partial interviews, refusals, non-contacts, and an estimated proportion of cases of unknown eligibility that are likely to be eligible. For pre-existing web panels, we report a composite response rate that combines the initial panel recruitment rate in the country with the response rate for the specific PVS sample. In countries using dual-mode data collection, separate response rates are provided for in-person and telephone interviews.

**Table B GDP per capita, self-reported mental health status, and care for mental health across 18 countries**

|  | **Ethiopia** | **Kenya** | **Nigeria** | **South Africa** | **Peru** | **Colombia** | **Mexico** | **Uruguay** | **Argentina** | **Lao PDR** | **India** | **China** | **Rep. of Korea** | **Romania** | **Greece** | **Italy** | **United Kingdom** | **United States** | **Total** |
| --- | --- | --- | --- | --- | --- | --- | --- | --- | --- | --- | --- | --- | --- | --- | --- | --- | --- | --- | --- |
|  | (N = 2779) | (N = 2305) | (N = 2555) | (N = 2036) | (N = 1255) | (N = 1237) | (N = 1002) | (N = 1237) | (N = 1190) | (N = 2007) | (N = 2004) | (N = 2625) | (N = 2000) | (N = 2001) | (N = 2008) | (N = 1001) | (N = 1677) | (N = 1500) | (N = 32419) |
| **GDP per capita (PPP)** | 3058 | 6307 | 6207 | 15194 | 16974 | 20784 | 24790 | 34427 | 30082 | 9292 | 10166 | 24569 | 52204 | 45777 | 41182 | 57801 | 58225 | 82769 | - |
| **Self-rated mental health** | | | | | | | | | | | | | | | | | | | |
| Good mental health | 2158 (77.6%) | 2128 (92.4%) | 2435 (95.3%) | 1642 (80.7%) | 923 (73.5%) | 1052 (85.3%) | 772 (77.0%) | 1057 (85.6%) | 1021 (85.9%) | 1517 (75.8%) | 1349 (67.3%) | 1586 (60.4%) | 1512 (75.6%) | 1809 (90.4%) | 1721 (85.6%) | 885 (88.4%) | 1265 (75.6%) | 1246 (83.2%) | 26068 (80.5%) |
| Poor mental health | 622 (22.4%) | 175 (7.6%) | 121 (4.7%) | 394 (19.3%) | 333 (26.5%) | 182 (14.7%) | 231 (23.0%) | 178 (14.4%) | 168 (14.1%) | 484 (24.2%) | 655 (32.7%) | 1040 (39.6%) | 490 (24.4%) | 193 (9.6%) | 290 (14.4%) | 117 (11.6%) | 408 (24.4%) | 252 (16.8%) | 6324 (19.5%) |
| **Received care for depression, anxiety, or another mental health condition** | | | | | | | | | | | | | | | | | | | |
| No | 2486 (92.9%) | 2235 (97.4%) | 2421 (95.1%) | 1753 (86.1%) | 1126 (89.7%) | 1090 (88.2%) | 848 (84.9%) | 1055 (85.3%) | 1014 (85.3%) | 1995 (99.3%) | 1807 (91.0%) | 2458 (93.6%) | 1634 (83.0%) | 1837 (91.9%) | 1811 (90.1%) | 919 (91.9%) | 1329 (80.4%) | 1162 (78.1%) | 28973 (90.0%) |
| Yes | 191 (7.1%) | 59 (2.6%) | 126 (4.9%) | 284 (13.9%) | 129 (10.3%) | 146 (11.8%) | 151 (15.1%) | 182 (14.7%) | 175 (14.7%) | 14 (0.7%) | 180 (9.0%) | 168 (6.4%) | 336 (17.0%) | 162 (8.1%) | 199 (9.9%) | 81 (8.1%) | 324 (19.6%) | 325 (21.9%) | 3224 (10.0%) |
| **Poor mental health and received care (among respondents with poor/fair mental health)** | | | | | | | | | | | | | | | | | | | |
| Poor mental health and did not receive care | 478 (91.6%) | 149 (97.4%) | 102 (96.6%) | 242 (70.4%) | 240 (82.5%) | 118 (74.4%) | 148 (73.4%) | 91 (58.3%) | 92 (62.8%) | 419 (99.1%) | 507 (89.6%) | 840 (92.6%) | 309 (74.2%) | 130 (77.4%) | 173 (68.5%) | 72 (70.1%) | 167 (47.6%) | 110 (50.7%) | 4377 (80.0%) |
| Poor mental health and received care | 44 (8.4%) | 4 (2.6%) | 4 (3.4%) | 102 (29.6%) | 51 (17.5%) | 41 (25.6%) | 54 (26.6%) | 65 (41.7%) | 55 (37.2%) | 4 (0.9%) | 59 (10.4%) | 68 (7.4%) | 108 (25.8%) | 39 (22.6%) | 80 (31.5%) | 31 (29.9%) | 184 (52.4%) | 107 (49.3%) | 1092 (20.0%) |
| **Mental health and care combinations (full sample)** | | | | | | | | | | | | | | | | | | | |
| Poor mental health and did not receive care | 548 (20.4%) | 170 (7.4%) | 117 (4.6%) | 278 (13.6%) | 275 (21.8%) | 135 (10.9%) | 170 (17.0%) | 104 (8.4%) | 105 (8.8%) | 480 (23.9%) | 581 (29.3%) | 963 (36.6%) | 354 (17.9%) | 149 (7.4%) | 199 (9.9%) | 82 (8.2%) | 192 (11.6%) | 125 (8.4%) | 5018 (15.6%) |
| Poor mental health and received care | 51 (1.9%) | 5 (0.2%) | 5 (0.2%) | 117 (5.7%) | 59 (4.6%) | 47 (3.7%) | 62 (6.1%) | 74 (6.0%) | 62 (5.2%) | 5 (0.2%) | 68 (3.4%) | 78 (2.9%) | 124 (6.2%) | 44 (2.2%) | 92 (4.5%) | 35 (3.5%) | 211 (12.7%) | 122 (8.2%) | 1252 (3.9%) |
| Good mental health and did not receive care | 1941 (72.5%) | 2064 (90.0%) | 2306 (90.5%) | 1477 (72.5%) | 853 (67.9%) | 955 (77.5%) | 679 (68.0%) | 952 (77.1%) | 909 (76.5%) | 1510 (75.4%) | 1226 (61.7%) | 1497 (57.0%) | 1281 (65.0%) | 1690 (84.5%) | 1614 (80.3%) | 838 (83.8%) | 1137 (68.8%) | 1035 (69.7%) | 23955 (74.4%) |
| Good mental health and received care | 140 (5.2%) | 55 (2.4%) | 122 (4.8%) | 167 (8.2%) | 71 (5.7%) | 98 (7.9%) | 90 (8.9%) | 106 (8.6%) | 113 (9.5%) | 9 (0.4%) | 113 (5.6%) | 91 (3.4%) | 213 (10.8%) | 118 (5.9%) | 108 (5.4%) | 46 (4.6%) | 114 (6.9%) | 204 (13.7%) | 1971 (6.1%) |

GDP per capita is shown in purchasing power parities (PPPs), which adjust for price differences across countries to allow for more accurate comparisons of economic output and living standards. All Ns and percentages are survey‑weighted to be nationally representative within each country.

**Table C Demographic factors, health status, healthcare utilization, and quality of care by mental health status across 18 countries**

|  | | | **Gender** | | **Age** | | | **Self-rated health** | | **Chronic illness: longstanding illness or health problem** | | **Patient activation: manage overall health and tell a provider concerns** | |
| --- | --- | --- | --- | --- | --- | --- | --- | --- | --- | --- | --- | --- | --- |
|  |  |  | **Male** | **Female or another gender** | **18-29** | **30-49** | **50+** | **Poor or Fair** | **Good, Very good, or Excellent** | **No** | **Yes** | **Not activated** | **Activated** |
| **Ethiopia** | **Poor mental health** | (N = 477) | 292 (46.8%) | 331 (53.2%) | 181 (29.1%) | 228 (36.6%) | 214 (34.3%) | 424 (68.0%) | 199 (32.0%) | 483 (77.7%) | 139 (22.3%) | 387 (62.1%) | 236 (37.9%) |
|  | **Good mental health** | (N = 2302) | 1100 (51.0%) | 1058 (49.0%) | 982 (45.5%) | 898 (41.6%) | 278 (12.8%) | 430 (19.9%) | 1728 (80.1%) | 1932 (89.5%) | 226 (10.5%) | 852 (39.5%) | 1306 (60.5%) |
|  | **Total** | (N = 2779) | 1392 (50.1%) | 1388 (49.9%) | 1163 (41.8%) | 1126 (40.5%) | 491 (17.7%) | 853 (30.7%) | 1927 (69.3%) | 2415 (86.9%) | 365 (13.1%) | 1239 (44.6%) | 1541 (55.4%) |
| **Kenya** | **Poor mental health** | (N = 121) | 64 (36.7%) | 111 (63.3%) | 46 (25.9%) | 69 (39.1%) | 61 (35.0%) | 108 (61.5%) | 68 (38.5%) | 120 (68.4%) | 55 (31.6%) | 101 (57.5%) | 74 (42.5%) |
|  | **Good mental health** | (N = 2179) | 1076 (50.6%) | 1053 (49.4%) | 906 (42.7%) | 883 (41.6%) | 333 (15.7%) | 320 (15.1%) | 1803 (84.9%) | 1822 (85.6%) | 307 (14.4%) | 991 (46.5%) | 1138 (53.5%) |
|  | **Total** | (N = 2305) | 1142 (49.5%) | 1164 (50.5%) | 952 (41.4%) | 952 (41.4%) | 395 (17.2%) | 428 (18.6%) | 1872 (81.4%) | 1944 (84.3%) | 362 (15.7%) | 1092 (47.4%) | 1214 (52.6%) |
| **Nigeria** | **Poor mental health** | (N = 91) | 56 (46.1%) | 66 (53.9%) | 24 (19.4%) | 36 (29.7%) | 62 (50.8%) | 65 (54.1%) | 56 (45.9%) | 47 (38.8%) | 74 (61.2%) | 67 (54.9%) | 55 (45.1%) |
|  | **Good mental health** | (N = 2464) | 1240 (50.9%) | 1195 (49.1%) | 994 (41.1%) | 1053 (43.6%) | 369 (15.3%) | 43 (1.8%) | 2392 (98.2%) | 2188 (89.9%) | 246 (10.1%) | 1219 (50.1%) | 1216 (49.9%) |
|  | **Total** | (N = 2555) | 1296 (50.7%) | 1260 (49.3%) | 1017 (40.1%) | 1089 (42.9%) | 430 (16.9%) | 108 (4.2%) | 2447 (95.8%) | 2235 (87.5%) | 320 (12.5%) | 1286 (50.3%) | 1270 (49.7%) |
| **South Africa** | **Poor mental health** | (N = 317) | 168 (42.6%) | 226 (57.4%) | 98 (24.8%) | 168 (42.5%) | 129 (32.7%) | 260 (66.0%) | 134 (34.0%) | 232 (58.7%) | 163 (41.3%) | 237 (60.2%) | 157 (39.8%) |
|  | **Good mental health** | (N = 1717) | 824 (50.2%) | 819 (49.8%) | 504 (30.7%) | 771 (47.0%) | 368 (22.4%) | 356 (21.7%) | 1286 (78.3%) | 1250 (76.2%) | 392 (23.8%) | 648 (39.4%) | 995 (60.6%) |
|  | **Total** | (N = 2036) | 992 (48.7%) | 1045 (51.3%) | 602 (29.5%) | 939 (46.1%) | 497 (24.4%) | 616 (30.2%) | 1421 (69.8%) | 1481 (72.8%) | 554 (27.2%) | 885 (43.4%) | 1152 (56.6%) |
| **Peru** | **Poor mental health** | (N = 279) | 151 (45.3%) | 182 (54.7%) | 80 (23.8%) | 134 (40.3%) | 120 (35.9%) | 260 (78.2%) | 73 (21.8%) | 206 (61.8%) | 127 (38.2%) | 266 (79.9%) | 67 (20.1%) |
|  | **Good mental health** | (N = 976) | 467 (50.6%) | 457 (49.4%) | 249 (26.9%) | 395 (42.8%) | 280 (30.3%) | 302 (32.7%) | 622 (67.3%) | 737 (79.9%) | 186 (20.1%) | 585 (63.3%) | 339 (36.7%) |
|  | **Total** | (N = 1255) | 618 (49.2%) | 638 (50.8%) | 328 (26.1%) | 529 (42.1%) | 399 (31.7%) | 562 (44.7%) | 694 (55.3%) | 942 (75.1%) | 313 (24.9%) | 850 (67.7%) | 406 (32.3%) |
| **Colombia** | **Poor mental health** | (N = 174) | 56 (30.6%) | 127 (69.4%) | 53 (29.2%) | 59 (32.5%) | 70 (38.4%) | 109 (59.6%) | 74 (40.4%) | 97 (53.0%) | 86 (47.0%) | 129 (71.3%) | 52 (28.7%) |
|  | **Good mental health** | (N = 1060) | 537 (51.0%) | 516 (49.0%) | 297 (28.2%) | 403 (38.3%) | 353 (33.5%) | 222 (21.1%) | 829 (78.9%) | 803 (76.3%) | 249 (23.7%) | 523 (49.8%) | 528 (50.2%) |
|  | **Total** | (N = 1237) | 594 (48.0%) | 644 (52.0%) | 350 (28.3%) | 463 (37.4%) | 425 (34.3%) | 331 (26.7%) | 906 (73.3%) | 902 (72.9%) | 335 (27.1%) | 655 (53.0%) | 580 (47.0%) |
| **Mexico** | **Poor mental health** | (N = 203) | 70 (30.2%) | 162 (69.8%) | 44 (18.9%) | 75 (32.3%) | 113 (48.8%) | 186 (81.1%) | 44 (18.9%) | 147 (63.5%) | 85 (36.5%) | 190 (82.0%) | 42 (18.0%) |
|  | **Good mental health** | (N = 799) | 403 (52.2%) | 369 (47.8%) | 235 (30.4%) | 327 (42.3%) | 211 (27.3%) | 217 (28.2%) | 552 (71.8%) | 620 (80.5%) | 151 (19.5%) | 383 (49.7%) | 388 (50.3%) |
|  | **Total** | (N = 1002) | 473 (47.1%) | 530 (52.9%) | 279 (27.8%) | 401 (40.0%) | 324 (32.3%) | 402 (40.3%) | 595 (59.7%) | 767 (76.6%) | 235 (23.4%) | 572 (57.2%) | 429 (42.8%) |
| **Uruguay** | **Poor mental health** | (N = 156) | 50 (28.2%) | 128 (71.8%) | 50 (27.9%) | 70 (39.0%) | 59 (33.1%) | 88 (49.5%) | 90 (50.5%) | 87 (48.9%) | 91 (51.1%) | 108 (61.1%) | 69 (38.9%) |
|  | **Good mental health** | (N = 1078) | 536 (50.7%) | 522 (49.3%) | 217 (20.6%) | 381 (36.1%) | 458 (43.4%) | 200 (19.0%) | 853 (81.0%) | 616 (58.2%) | 442 (41.8%) | 417 (39.8%) | 631 (60.2%) |
|  | **Total** | (N = 1237) | 588 (47.5%) | 650 (52.5%) | 267 (21.6%) | 451 (36.5%) | 519 (42.0%) | 291 (23.5%) | 943 (76.5%) | 703 (56.8%) | 535 (43.2%) | 528 (43.0%) | 700 (57.0%) |
| **Argentina** | **Poor mental health** | (N = 161) | 62 (36.8%) | 106 (63.2%) | 27 (15.7%) | 75 (44.6%) | 67 (39.7%) | 88 (52.4%) | 80 (47.6%) | 81 (48.2%) | 87 (51.8%) | 102 (61.2%) | 65 (38.8%) |
|  | **Good mental health** | (N = 1027) | 398 (39.0%) | 623 (61.0%) | 101 (9.8%) | 457 (44.8%) | 464 (45.4%) | 185 (18.1%) | 834 (81.9%) | 614 (60.2%) | 406 (39.8%) | 418 (40.9%) | 603 (59.1%) |
|  | **Total** | (N = 1190) | 462 (38.7%) | 729 (61.3%) | 127 (10.7%) | 533 (44.8%) | 531 (44.6%) | 273 (22.9%) | 915 (77.1%) | 696 (58.6%) | 492 (41.4%) | 522 (43.9%) | 668 (56.1%) |
| **Lao PDR** | **Poor mental health** | (N = 477) | 208 (43.0%) | 276 (57.0%) | 171 (35.3%) | 213 (44.0%) | 100 (20.7%) | 353 (72.9%) | 131 (27.1%) | 327 (67.6%) | 157 (32.4%) | 331 (68.4%) | 153 (31.6%) |
|  | **Good mental health** | (N = 1527) | 765 (50.4%) | 752 (49.6%) | 454 (29.9%) | 626 (41.3%) | 437 (28.8%) | 229 (15.1%) | 1288 (84.9%) | 1197 (79.0%) | 318 (21.0%) | 586 (38.7%) | 930 (61.3%) |
|  | **Total** | (N = 2007) | 972 (48.4%) | 1035 (51.6%) | 626 (31.2%) | 840 (41.8%) | 543 (27.0%) | 587 (29.2%) | 1421 (70.8%) | 1526 (76.1%) | 480 (23.9%) | 923 (46.0%) | 1083 (54.0%) |
| **India** | **Poor mental health** | (N = 604) | 381 (58.2%) | 274 (41.8%) | 202 (30.9%) | 280 (42.7%) | 174 (26.5%) | 476 (72.7%) | 179 (27.3%) | 519 (79.5%) | 134 (20.5%) | 405 (61.9%) | 249 (38.1%) |
|  | **Good mental health** | (N = 1397) | 661 (49.0%) | 688 (51.0%) | 487 (36.1%) | 527 (39.0%) | 336 (24.9%) | 304 (22.5%) | 1045 (77.5%) | 1190 (88.3%) | 158 (11.7%) | 778 (57.8%) | 567 (42.2%) |
|  | **Total** | (N = 2004) | 1043 (52.0%) | 961 (48.0%) | 691 (34.4%) | 806 (40.2%) | 509 (25.4%) | 781 (39.0%) | 1224 (61.0%) | 1711 (85.4%) | 292 (14.6%) | 1184 (59.2%) | 816 (40.8%) |
| **China** | **Poor mental health** | (N = 879) | 556 (53.5%) | 484 (46.5%) | 152 (14.6%) | 309 (29.7%) | 579 (55.7%) | 897 (86.2%) | 144 (13.8%) | 676 (65.0%) | 365 (35.0%) | 741 (71.2%) | 299 (28.8%) |
|  | **Good mental health** | (N = 1746) | 787 (49.6%) | 799 (50.4%) | 309 (19.5%) | 708 (44.6%) | 570 (35.9%) | 526 (33.2%) | 1060 (66.8%) | 1405 (88.6%) | 182 (11.4%) | 684 (43.1%) | 903 (56.9%) |
|  | **Total** | (N = 2625) | 1343 (51.2%) | 1283 (48.8%) | 461 (17.5%) | 1016 (38.7%) | 1149 (43.8%) | 1422 (54.2%) | 1204 (45.8%) | 2080 (79.2%) | 546 (20.8%) | 1424 (54.2%) | 1202 (45.8%) |
| **Rep. of Korea** | **Poor mental health** | (N = 489) | 227 (46.4%) | 262 (53.6%) | 87 (17.8%) | 188 (38.4%) | 214 (43.8%) | 392 (80.2%) | 97 (19.8%) | 216 (44.2%) | 273 (55.8%) | 465 (95.1%) | 24 (4.9%) |
|  | **Good mental health** | (N = 1511) | 766 (50.7%) | 745 (49.3%) | 247 (16.3%) | 479 (31.7%) | 785 (52.0%) | 425 (28.1%) | 1086 (71.9%) | 999 (66.1%) | 512 (33.9%) | 1356 (89.7%) | 155 (10.3%) |
|  | **Total** | (N = 2000) | 993 (49.6%) | 1007 (50.3%) | 334 (16.7%) | 667 (33.4%) | 999 (50.0%) | 817 (40.8%) | 1183 (59.2%) | 1215 (60.8%) | 785 (39.2%) | 1821 (91.0%) | 179 (8.9%) |
| **Romania** | **Poor mental health** | (N = 107) | 47 (24.1%) | 147 (75.9%) | 12 (6.1%) | 50 (25.5%) | 132 (68.4%) | 167 (86.3%) | 27 (13.7%) | 51 (26.4%) | 143 (73.6%) | 131 (69.1%) | 59 (30.9%) |
|  | **Good mental health** | (N = 1894) | 916 (50.6%) | 893 (49.4%) | 295 (16.3%) | 672 (37.1%) | 843 (46.6%) | 576 (31.9%) | 1233 (68.1%) | 1155 (63.9%) | 654 (36.1%) | 1016 (56.2%) | 793 (43.8%) |
|  | **Total** | (N = 2001) | 962 (48.1%) | 1040 (51.9%) | 306 (15.3%) | 721 (36.0%) | 975 (48.7%) | 743 (37.1%) | 1259 (62.9%) | 1206 (60.2%) | 796 (39.8%) | 1147 (57.4%) | 852 (42.6%) |
| **Greece** | **Poor mental health** | (N = 225) | 87 (30.0%) | 203 (70.0%) | 34 (11.5%) | 81 (27.8%) | 176 (60.6%) | 168 (57.8%) | 122 (42.2%) | 125 (43.2%) | 165 (56.8%) | 200 (69.2%) | 90 (30.8%) |
|  | **Good mental health** | (N = 1783) | 889 (51.7%) | 831 (48.3%) | 323 (18.8%) | 634 (36.9%) | 763 (44.3%) | 238 (13.8%) | 1482 (86.2%) | 1223 (71.2%) | 496 (28.8%) | 1050 (61.1%) | 669 (38.9%) |
|  | **Total** | (N = 2008) | 976 (48.6%) | 1033 (51.4%) | 357 (17.7%) | 715 (35.6%) | 938 (46.7%) | 405 (20.2%) | 1604 (79.8%) | 1348 (67.1%) | 660 (32.9%) | 1250 (62.3%) | 758 (37.7%) |
| **Italy** | **Poor mental health** | (N = 102) | 52 (44.5%) | 65 (55.5%) | 20 (16.6%) | 23 (19.6%) | 75 (63.8%) | 65 (55.1%) | 53 (44.9%) | 71 (60.9%) | 46 (39.1%) | 109 (93.0%) | 9 (7.0%) |
|  | **Good mental health** | (N = 899) | 429 (48.5%) | 456 (51.5%) | 120 (13.5%) | 279 (31.5%) | 487 (55.0%) | 248 (27.9%) | 638 (72.1%) | 608 (68.7%) | 277 (31.3%) | 700 (79.1%) | 185 (20.9%) |
|  | **Total** | (N = 1001) | 481 (48.0%) | 521 (52.0%) | 139 (13.9%) | 302 (30.1%) | 561 (56.0%) | 312 (31.1%) | 690 (68.9%) | 678 (67.8%) | 323 (32.2%) | 809 (80.7%) | 193 (19.3%) |
| **United Kingdom** | **Poor mental health** | (N = 397) | 181 (44.2%) | 228 (55.8%) | 111 (27.0%) | 149 (36.5%) | 150 (36.5%) | 205 (50.4%) | 201 (49.6%) | 105 (26.1%) | 297 (73.9%) | 341 (83.6%) | 68 (16.4%) |
|  | **Good mental health** | (N = 1277) | 628 (49.7%) | 637 (50.3%) | 200 (15.8%) | 407 (32.1%) | 659 (52.1%) | 207 (16.3%) | 1058 (83.7%) | 706 (56.5%) | 543 (43.5%) | 739 (58.5%) | 524 (41.5%) |
|  | **Total** | (N = 1677) | 810 (48.3%) | 868 (51.7%) | 310 (18.5%) | 556 (33.1%) | 812 (48.4%) | 413 (24.7%) | 1260 (75.3%) | 811 (49.1%) | 841 (50.9%) | 1081 (64.7%) | 591 (35.3%) |
| **United States** | **Poor mental health** | (N = 251) | 75 (29.7%) | 177 (70.3%) | 99 (39.3%) | 97 (38.2%) | 57 (22.5%) | 99 (39.2%) | 154 (60.8%) | 92 (36.4%) | 161 (63.6%) | 199 (78.9%) | 53 (21.1%) |
|  | **Good mental health** | (N = 1248) | 652 (52.3%) | 595 (47.7%) | 205 (16.4%) | 406 (32.5%) | 637 (51.1%) | 149 (11.9%) | 1098 (88.1%) | 796 (63.8%) | 451 (36.2%) | 569 (45.6%) | 678 (54.4%) |
|  | **Total** | (N = 1500) | 729 (48.6%) | 772 (51.4%) | 304 (20.2%) | 502 (33.4%) | 696 (46.3%) | 248 (16.5%) | 1253 (83.5%) | 890 (59.3%) | 611 (40.7%) | 768 (51.2%) | 733 (48.8%) |
| **All countries** | **Poor mental health** | (N = 5510) | 2777 (43.9%) | 3547 (56.1%) | 1484 (23.5%) | 2296 (36.3%) | 2545 (40.2%) | 4401 (69.6%) | 1918 (30.4%) | 3674 (58.2%) | 2640 (41.8%) | 4503 (71.3%) | 1814 (28.7%) |
|  | **Good mental health** | (N = 26884) | 13068 (50.1%) | 13000 (49.9%) | 7118 (27.3%) | 10299 (39.6%) | 8623 (33.1%) | 5172 (19.9%) | 20878 (80.1%) | 19854 (76.2%) | 6189 (23.8%) | 13507 (51.9%) | 12540 (48.1%) |
|  | **Total** | (N = 32419) | 15857 (48.9%) | 16561 (51.1%) | 8605 (26.6%) | 12601 (38.9%) | 11185 (34.5%) | 9583 (29.6%) | 22808 (70.4%) | 23542 (72.7%) | 8838 (27.3%) | 18028 (55.7%) | 14359 (44.3%) |

**Table C (continued) Demographic factors, health status, healthcare utilization, and quality of care by mental health status across 18 countries**

|  | | | **Usual source of care** | | **Unmet need for care** | | **Overall quality rating of last visit to a healthcare provider** | | **Discriminated against** | | **Public primary care system rating for: mental health** | |
| --- | --- | --- | --- | --- | --- | --- | --- | --- | --- | --- | --- | --- |
|  |  |  | **No** | **Yes** | **No** | **Yes** | **Poor or Fair** | **Good, Very good, or Excellent** | **No** | **Yes** | **Poor or Fair** | **Good, Very good, or Excellent** |
| **Ethiopia** | **Poor mental health** | (N = 477) | 154 (24.7%) | 469 (75.3%) | 530 (85.2%) | 93 (14.8%) | 248 (62.5%) | 149 (37.5%) | 350 (85.7%) | 59 (14.3%) | 440 (80.5%) | 107 (19.5%) |
|  | **Good mental health** | (N = 2302) | 635 (29.4%) | 1523 (70.6%) | 1945 (90.2%) | 213 (9.8%) | 708 (54.9%) | 581 (45.1%) | 1181 (87.5%) | 170 (12.5%) | 1442 (80.1%) | 358 (19.9%) |
|  | **Total** | (N = 2779) | 789 (28.4%) | 1991 (71.6%) | 2475 (89.0%) | 305 (11.0%) | 955 (56.7%) | 729 (43.3%) | 1531 (87.1%) | 228 (12.9%) | 1881 (80.2%) | 464 (19.8%) |
| **Kenya** | **Poor mental health** | (N = 121) | 40 (22.8%) | 135 (77.2%) | 138 (79.2%) | 37 (20.8%) | 94 (62.1%) | 58 (37.9%) | 138 (86.5%) | 22 (13.5%) | 98 (73.6%) | 35 (26.4%) |
|  | **Good mental health** | (N = 2179) | 667 (31.3%) | 1462 (68.7%) | 1672 (78.6%) | 456 (21.4%) | 934 (57.0%) | 704 (43.0%) | 1476 (89.5%) | 173 (10.5%) | 1266 (73.7%) | 452 (26.3%) |
|  | **Total** | (N = 2305) | 707 (30.7%) | 1598 (69.3%) | 1812 (78.6%) | 492 (21.4%) | 1029 (57.5%) | 762 (42.5%) | 1616 (89.3%) | 195 (10.7%) | 1364 (73.7%) | 487 (26.3%) |
| **Nigeria** | **Poor mental health** | (N = 91) | 16 (13.1%) | 105 (86.9%) | 68 (56.2%) | 53 (43.8%) | 64 (60.0%) | 43 (40.0%) | 69 (64.5%) | 38 (35.5%) | 56 (86.3%) | 9 (13.7%) |
|  | **Good mental health** | (N = 2464) | 699 (28.7%) | 1737 (71.3%) | 2233 (91.8%) | 201 (8.2%) | 352 (21.4%) | 1292 (78.6%) | 1598 (90.8%) | 163 (9.2%) | 1268 (64.5%) | 699 (35.5%) |
|  | **Total** | (N = 2555) | 714 (27.9%) | 1842 (72.1%) | 2301 (90.1%) | 253 (9.9%) | 415 (23.7%) | 1334 (76.3%) | 1666 (89.3%) | 200 (10.7%) | 1323 (65.2%) | 708 (34.8%) |
| **South Africa** | **Poor mental health** | (N = 317) | 102 (26.2%) | 286 (73.8%) | 346 (87.8%) | 49 (12.2%) | 173 (53.7%) | 149 (46.3%) | 304 (90.5%) | 32 (9.5%) | 293 (80.1%) | 73 (19.9%) |
|  | **Good mental health** | (N = 1717) | 558 (34.0%) | 1081 (66.0%) | 1498 (91.2%) | 145 (8.8%) | 524 (43.5%) | 682 (56.5%) | 1119 (89.0%) | 139 (11.0%) | 1012 (67.4%) | 490 (32.6%) |
|  | **Total** | (N = 2036) | 660 (32.5%) | 1366 (67.5%) | 1843 (90.5%) | 194 (9.5%) | 697 (45.6%) | 831 (54.4%) | 1422 (89.3%) | 171 (10.7%) | 1305 (69.9%) | 562 (30.1%) |
| **Peru** | **Poor mental health** | (N = 279) | 76 (22.6%) | 258 (77.4%) | 208 (62.5%) | 125 (37.5%) | 160 (62.4%) | 96 (37.6%) | 226 (83.2%) | 46 (16.8%) | 297 (89.8%) | 34 (10.2%) |
|  | **Good mental health** | (N = 976) | 223 (24.2%) | 699 (75.8%) | 724 (78.5%) | 199 (21.5%) | 476 (68.1%) | 223 (31.9%) | 658 (86.6%) | 102 (13.4%) | 812 (89.9%) | 92 (10.1%) |
|  | **Total** | (N = 1255) | 298 (23.7%) | 956 (76.3%) | 931 (74.2%) | 323 (25.8%) | 636 (66.6%) | 319 (33.4%) | 884 (85.7%) | 148 (14.3%) | 1108 (89.9%) | 125 (10.1%) |
| **Colombia** | **Poor mental health** | (N = 174) | 43 (23.6%) | 139 (76.4%) | 104 (57.1%) | 78 (42.9%) | 121 (73.9%) | 43 (26.1%) | 143 (86.5%) | 23 (13.5%) | 150 (86.4%) | 24 (13.6%) |
|  | **Good mental health** | (N = 1060) | 228 (21.6%) | 825 (78.4%) | 883 (84.0%) | 169 (16.0%) | 536 (62.1%) | 327 (37.9%) | 852 (94.3%) | 52 (5.7%) | 807 (82.9%) | 167 (17.1%) |
|  | **Total** | (N = 1237) | 270 (21.8%) | 968 (78.2%) | 991 (80.1%) | 247 (19.9%) | 657 (63.8%) | 374 (36.2%) | 999 (93.1%) | 74 (6.9%) | 957 (83.1%) | 194 (16.9%) |
| **Mexico** | **Poor mental health** | (N = 203) | 45 (19.1%) | 187 (80.9%) | 201 (87.2%) | 30 (12.8%) | 109 (59.1%) | 75 (40.9%) | 175 (92.5%) | 15 (7.5%) | 182 (86.6%) | 29 (13.4%) |
|  | **Good mental health** | (N = 799) | 138 (17.8%) | 634 (82.2%) | 733 (95.1%) | 38 (4.9%) | 284 (47.2%) | 317 (52.8%) | 563 (92.9%) | 44 (7.1%) | 599 (84.3%) | 112 (15.7%) |
|  | **Total** | (N = 1002) | 182 (18.1%) | 821 (81.9%) | 934 (93.3%) | 68 (6.7%) | 392 (50.0%) | 392 (50.0%) | 737 (92.8%) | 58 (7.2%) | 780 (84.8%) | 140 (15.2%) |
| **Uruguay** | **Poor mental health** | (N = 156) | 12 (6.8%) | 164 (93.2%) | 129 (72.5%) | 49 (27.5%) | 103 (60.7%) | 67 (39.3%) | 144 (83.5%) | 29 (16.5%) | 137 (82.7%) | 29 (17.3%) |
|  | **Good mental health** | (N = 1078) | 65 (6.1%) | 989 (93.9%) | 957 (90.5%) | 101 (9.5%) | 331 (36.3%) | 582 (63.7%) | 880 (93.3%) | 64 (6.7%) | 786 (84.4%) | 146 (15.6%) |
|  | **Total** | (N = 1237) | 77 (6.2%) | 1155 (93.8%) | 1088 (87.9%) | 150 (12.1%) | 433 (40.0%) | 650 (60.0%) | 1026 (91.8%) | 92 (8.2%) | 924 (84.1%) | 175 (15.9%) |
| **Argentina** | **Poor mental health** | (N = 161) | 32 (18.7%) | 136 (81.3%) | 112 (66.8%) | 56 (33.2%) | 56 (38.9%) | 88 (61.1%) | 114 (77.4%) | 34 (22.6%) | 118 (85.8%) | 20 (14.2%) |
|  | **Good mental health** | (N = 1027) | 165 (16.1%) | 857 (83.9%) | 842 (82.5%) | 179 (17.5%) | 295 (33.4%) | 589 (66.6%) | 797 (89.1%) | 97 (10.9%) | 577 (79.4%) | 150 (20.6%) |
|  | **Total** | (N = 1190) | 197 (16.5%) | 993 (83.5%) | 956 (80.3%) | 234 (19.7%) | 351 (34.1%) | 677 (65.9%) | 911 (87.5%) | 131 (12.5%) | 695 (80.4%) | 170 (19.6%) |
| **Lao PDR** | **Poor mental health** | (N = 477) | 41 (8.6%) | 434 (91.4%) | 410 (84.8%) | 74 (15.2%) | 221 (72.4%) | 84 (27.6%) | 239 (80.3%) | 59 (19.7%) | 356 (85.2%) | 62 (14.8%) |
|  | **Good mental health** | (N = 1527) | 179 (12.0%) | 1307 (88.0%) | 1254 (82.9%) | 259 (17.1%) | 719 (72.6%) | 272 (27.4%) | 857 (90.3%) | 92 (9.7%) | 1100 (90.4%) | 117 (9.6%) |
|  | **Total** | (N = 2007) | 227 (11.5%) | 1740 (88.5%) | 1670 (83.4%) | 333 (16.6%) | 939 (72.5%) | 357 (27.5%) | 1097 (88.0%) | 150 (12.0%) | 1462 (89.1%) | 179 (10.9%) |
| **India** | **Poor mental health** | (N = 604) | 354 (54.1%) | 300 (45.9%) | 594 (90.8%) | 61 (9.2%) | 238 (71.3%) | 96 (28.7%) | 388 (94.4%) | 23 (5.6%) | 371 (70.8%) | 153 (29.2%) |
|  | **Good mental health** | (N = 1397) | 673 (50.0%) | 674 (50.0%) | 1282 (95.4%) | 63 (4.6%) | 413 (55.5%) | 331 (44.5%) | 781 (97.0%) | 24 (3.0%) | 765 (73.6%) | 275 (26.4%) |
|  | **Total** | (N = 2004) | 1028 (51.3%) | 974 (48.7%) | 1878 (93.9%) | 123 (6.1%) | 651 (60.4%) | 426 (39.6%) | 1168 (96.2%) | 47 (3.8%) | 1137 (72.7%) | 428 (27.3%) |
| **China** | **Poor mental health** | (N = 879) | 487 (46.9%) | 553 (53.1%) | 967 (93.0%) | 73 (7.0%) | 604 (78.1%) | 170 (21.9%) | 597 (95.5%) | 28 (4.5%) | 784 (85.6%) | 133 (14.4%) |
|  | **Good mental health** | (N = 1746) | 660 (41.6%) | 927 (58.4%) | 1547 (97.6%) | 39 (2.4%) | 669 (65.3%) | 355 (34.7%) | 864 (97.3%) | 24 (2.7%) | 1112 (76.8%) | 336 (23.2%) |
|  | **Total** | (N = 2625) | 1147 (43.7%) | 1479 (56.3%) | 2514 (95.7%) | 112 (4.3%) | 1273 (70.8%) | 525 (29.2%) | 1460 (96.6%) | 52 (3.4%) | 1895 (80.2%) | 468 (19.8%) |
| **Korea** | **Poor mental health** | (N = 489) | 201 (41.1%) | 288 (58.9%) | 451 (92.2%) | 38 (7.8%) | 370 (79.6%) | 95 (20.4%) | 433 (93.1%) | 32 (6.9%) | 401 (82.0%) | 88 (18.0%) |
|  | **Good mental health** | (N = 1511) | 541 (35.8%) | 970 (64.2%) | 1430 (94.6%) | 81 (5.4%) | 969 (67.7%) | 462 (32.3%) | 1343 (93.9%) | 87 (6.1%) | 1165 (77.1%) | 346 (22.9%) |
|  | **Total** | (N = 2000) | 742 (37.1%) | 1258 (62.9%) | 1881 (94.0%) | 119 (5.9%) | 1339 (70.6%) | 557 (29.4%) | 1776 (93.7%) | 119 (6.3%) | 1566 (78.3%) | 434 (21.7%) |
| **Romania** | **Poor mental health** | (N = 107) | 21 (10.8%) | 173 (89.2%) | 159 (82.8%) | 34 (17.2%) | 83 (52.9%) | 74 (47.1%) | 132 (82.3%) | 29 (17.7%) | 139 (80.1%) | 35 (19.9%) |
|  | **Good mental health** | (N = 1894) | 292 (16.1%) | 1517 (83.9%) | 1634 (90.4%) | 175 (9.6%) | 512 (34.1%) | 986 (65.9%) | 1407 (92.6%) | 113 (7.4%) | 1101 (68.6%) | 505 (31.4%) |
|  | **Total** | (N = 2001) | 313 (15.6%) | 1689 (84.4%) | 1793 (89.6%) | 208 (10.4%) | 594 (35.9%) | 1060 (64.1%) | 1538 (91.6%) | 141 (8.4%) | 1240 (69.7%) | 540 (30.3%) |
| **Greece** | **Poor mental health** | (N = 225) | 124 (42.7%) | 166 (57.3%) | 239 (82.7%) | 51 (17.3%) | 108 (40.8%) | 156 (59.2%) | 225 (83.5%) | 45 (16.5%) | 161 (87.1%) | 24 (12.9%) |
|  | **Good mental health** | (N = 1783) | 851 (49.5%) | 869 (50.5%) | 1606 (93.4%) | 114 (6.6%) | 331 (23.2%) | 1091 (76.8%) | 1341 (94.0%) | 86 (6.0%) | 918 (87.8%) | 129 (12.2%) |
|  | **Total** | (N = 2008) | 974 (48.5%) | 1035 (51.5%) | 1845 (91.9%) | 164 (8.1%) | 438 (26.0%) | 1247 (74.0%) | 1565 (92.3%) | 130 (7.7%) | 1079 (87.7%) | 152 (12.3%) |
| **Italy** | **Poor mental health** | (N = 102) | 33 (27.8%) | 84 (72.2%) | 108 (92.5%) | 9 (7.5%) | 57 (56.6%) | 44 (43.4%) | 94 (92.0%) | 9 (8.0%) | 82 (83.3%) | 17 (16.7%) |
|  | **Good mental health** | (N = 899) | 221 (25.0%) | 663 (75.0%) | 831 (94.0%) | 54 (6.0%) | 256 (36.7%) | 441 (63.3%) | 644 (92.0%) | 57 (8.0%) | 556 (86.0%) | 91 (14.0%) |
|  | **Total** | (N = 1001) | 253 (25.3%) | 747 (74.7%) | 939 (93.8%) | 62 (6.2%) | 312 (39.2%) | 484 (60.8%) | 737 (92.0%) | 65 (8.0%) | 637 (85.7%) | 107 (14.3%) |
| **United Kingdom** | **Poor mental health** | (N = 397) | 57 (14.0%) | 347 (86.0%) | 268 (66.7%) | 134 (33.3%) | 162 (45.4%) | 194 (54.6%) | 329 (88.2%) | 44 (11.8%) | 310 (85.8%) | 52 (14.2%) |
|  | **Good mental health** | (N = 1277) | 149 (11.9%) | 1100 (88.1%) | 1011 (81.2%) | 235 (18.8%) | 306 (28.3%) | 775 (71.7%) | 1051 (95.1%) | 54 (4.9%) | 726 (80.2%) | 180 (19.8%) |
|  | **Total** | (N = 1677) | 205 (12.4%) | 1449 (87.6%) | 1279 (77.6%) | 369 (22.4%) | 467 (32.5%) | 970 (67.5%) | 1380 (93.4%) | 99 (6.6%) | 1036 (81.8%) | 231 (18.2%) |
| **United States** | **Poor mental health** | (N = 251) | 58 (22.9%) | 194 (77.1%) | 143 (56.9%) | 109 (43.1%) | 109 (48.1%) | 118 (51.9%) | 189 (82.5%) | 40 (17.5%) | 156 (77.5%) | 46 (22.5%) |
|  | **Good mental health** | (N = 1248) | 198 (15.8%) | 1049 (84.2%) | 1073 (86.1%) | 174 (13.9%) | 251 (21.8%) | 900 (78.2%) | 1094 (95.3%) | 54 (4.7%) | 677 (74.4%) | 233 (25.6%) |
|  | **Total** | (N = 1500) | 256 (17.0%) | 1245 (83.0%) | 1218 (81.2%) | 282 (18.8%) | 359 (26.0%) | 1020 (74.0%) | 1284 (93.2%) | 94 (6.8%) | 833 (74.8%) | 280 (25.2%) |
| **All countries** | **Poor mental health** | (N = 5510) | 1889 (30.0%) | 4410 (70.0%) | 5170 (81.9%) | 1144 (18.1%) | 3070 (63.2%) | 1791 (36.8%) | 4280 (87.7%) | 599 (12.3%) | 4524 (82.5%) | 962 (17.5%) |
|  | **Good mental health** | (N = 26884) | 7133 (27.4%) | 18874 (72.6%) | 23147 (88.9%) | 2885 (11.1%) | 8857 (44.8%) | 10903 (55.2%) | 18496 (92.1%) | 1587 (7.9%) | 16681 (77.4%) | 4869 (22.6%) |
|  | **Total** | (N = 32419) | 9032 (27.9%) | 23298 (72.1%) | 28338 (87.5%) | 4032 (12.5%) | 11929 (48.4%) | 12706 (51.6%) | 22789 (91.2%) | 2187 (8.8%) | 21214 (78.4%) | 5836 (21.6%) |

**Table D1 Association between poor mental health status and confidence in the health system, adjusted**

|  | **Confidence in getting and affording good care** | | **Health system getting better** | | **Health system needs minor changes** | |
| --- | --- | --- | --- | --- | --- | --- |
| *Poor mental health* | Coefficient (OR) | 95% CI | Coefficient (OR) | 95% CI | Coefficient (OR) | 95% CI |
| Ethiopia | 1.31* | [1.02,1.68] | 1.13 | [0.85,1.50] | 1.03 | [0.75,1.42] |
| Kenya | 0.59* | [0.38,0.91] | 1.04 | [0.66,1.64] | 0.84 | [0.50,1.41] |
| Nigeria | 0.47** | [0.28,0.78] | 0.62 | [0.36,1.05] | 0.73 | [0.37,1.46] |
| South Africa | 0.8 | [0.60,1.06] | 0.67** | [0.50,0.91] | 0.95 | [0.67,1.34] |
| Peru | 0.62* | [0.42,0.91] | 0.98 | [0.68,1.41] | 0.9 | [0.54,1.50] |
| Colombia | 0.48** | [0.30,0.79] | 0.78 | [0.51,1.21] | 0.95 | [0.55,1.64] |
| Mexico | 1.05 | [0.71,1.55] | 1.44 | [0.94,2.20] | 1.28 | [0.79,2.06] |
| Uruguay | 0.69 | [0.43,1.11] | 0.86 | [0.56,1.32] | 1.02 | [0.60,1.72] |
| Argentina | 0.65 | [0.42,1.02] | 0.78 | [0.50,1.21] | 0.83 | [0.49,1.41] |
| Lao PDR | 0.54*** | [0.41,0.71] | 0.81 | [0.58,1.14] | 0.92 | [0.71,1.20] |
| India | 1.2 | [0.93,1.54] | 1.21 | [0.94,1.57] | 1.08 | [0.84,1.40] |
| China | 0.67*** | [0.54,0.82] | 1.2 | [0.80,1.80] | 0.79* | [0.64,0.98] |
| Rep. of Korea | 0.49*** | [0.39,0.62] | 0.70** | [0.55,0.88] | 1.03 | [0.82,1.29] |
| Romania | 0.50* | [0.26,0.94] | 0.8 | [0.47,1.37] | 2.19** | [1.21,3.96] |
| Greece | 0.82 | [0.51,1.32] | 1.01 | [0.64,1.58] | 1.06 | [0.60,1.86] |
| Italy | 0.98 | [0.59,1.64] | 1.6 | [0.83,3.09] | 1.56 | [0.94,2.58] |
| United Kingdom | 0.79 | [0.59,1.06] | 0.76 | [0.38,1.52] | 0.7 | [0.46,1.06] |
| United States | 0.61** | [0.44,0.85] | 0.76 | [0.47,1.21] | 0.85 | [0.54,1.36] |

Exponentiated coefficients; 95% confidence intervals in brackets

* p < 0.05, ** p < 0.01, *** p < 0.001

P‑values are from two‑sided Wald tests of coefficients in survey‑weighted logistic regression models.

Adjusted odds ratios (with 95% confidence intervals) from 18 country-specific regression models showing the association between poor mental health status and confidence in the health system presented in Figure 7 forest plots. The regression outcomes include: (1) respondents' confidence in getting and affording good care (defined as responding "somewhat" or "very" confident to both getting good quality care and affording care), (2) belief that the health system is getting better, and (3) perception that the health system needs only minor changes. Poor mental health is defined as respondents reporting "poor" or "fair" mental health on a five-point self-rating scale. Models are adjusted for gender, age, education, income, patient activation, urban/rural residence, insurance status, presence of chronic illness, quality of usual source of care, and perceived government management of the COVID-19 pandemic.

**Table D2 Association between poor mental health status and confidence in the health system, unadjusted**

|  | **Confidence in getting and affording good care** | | **Health system getting better** | | **Health system needs minor changes** | |
| --- | --- | --- | --- | --- | --- | --- |
| *Poor mental health* | Coefficient (OR) | 95% CI | Coefficient (OR) | 95% CI | Coefficient (OR) | 95% CI |
| Ethiopia | 1.05 | [0.86,1.29] | 1.01 | [0.80,1.28] | 1.13 | [0.88,1.45] |
| Kenya | 0.58** | [0.39,0.85] | 0.77 | [0.51,1.15] | 1.00 | [0.64,1.57] |
| Nigeria | 0.36*** | [0.24,0.55] | 0.48** | [0.30,0.76] | 0.82 | [0.44,1.51] |
| South Africa | 0.63*** | [0.49,0.81] | 0.72* | [0.56,0.93] | 0.96 | [0.70,1.32] |
| Peru | 0.55*** | [0.39,0.78] | 0.84 | [0.60,1.16] | 0.97 | [0.62,1.53] |
| Colombia | 0.41*** | [0.27,0.63] | 0.63* | [0.43,0.93] | 0.90 | [0.57,1.43] |
| Mexico | 0.71* | [0.51,0.97] | 0.97 | [0.71,1.34] | 1.48* | [1.02,2.16] |
| Uruguay | 0.43*** | [0.28,0.65] | 0.72 | [0.50,1.04] | 0.63* | [0.41,0.98] |
| Argentina | 0.49*** | [0.33,0.74] | 0.71 | [0.48,1.05] | 0.84 | [0.53,1.34] |
| Lao PDR | 0.38*** | [0.31,0.48] | 0.77 | [0.58,1.01] | 0.85 | [0.68,1.06] |
| India | 0.87 | [0.70,1.07] | 1.11 | [0.89,1.37] | 0.90 | [0.73,1.12] |
| China | 0.46*** | [0.39,0.54] | 1.59** | [1.13,2.25] | 0.61*** | [0.52,0.72] |
| Rep. of Korea | 0.38*** | [0.30,0.46] | 0.54*** | [0.44,0.66] | 0.86 | [0.70,1.06] |
| Romania | 0.34*** | [0.20,0.58] | 0.74 | [0.48,1.17] | 2.29*** | [1.42,3.69] |
| Greece | 0.51** | [0.34,0.77] | 0.76 | [0.53,1.08] | 0.84 | [0.51,1.39] |
| Italy | 0.62* | [0.40,0.95] | 1.48 | [0.85,2.60] | 1.08 | [0.70,1.66] |
| United Kingdom | 0.61*** | [0.48,0.77] | 0.87 | [0.51,1.49] | 0.72 | [0.50,1.02] |
| United States | 0.34*** | [0.25,0.45] | 0.62* | [0.40,0.95] | 0.43*** | [0.28,0.64] |

Exponentiated coefficients; 95% confidence intervals in brackets

* p < 0.05, ** p < 0.01, *** p < 0.001

P‑values are from two‑sided Wald tests of coefficients in survey‑weighted logistic regression models.

Odds ratios (with 95% confidence intervals) from 18 country-specific regression models showing the association between poor mental health status and confidence in the health system. The regression outcomes include: (1) respondents' confidence in getting and affording good care (defined as responding "somewhat" or "very" confident to both getting good quality care and affording care), (2) belief that the health system is getting better, and (3) perception that the health system needs only minor changes. Poor mental health is defined as respondents reporting "poor" or "fair" mental health on a five-point self-rating scale. Models are unadjusted.

**Figure A Confidence in the health system for people in poor mental health, unadjusted**

Forest plots showing unadjusted odds of confidence in the health system (with 95% confidence intervals) among those with poor mental health status compared to those with good mental health across 18 countries. Panel A shows associations with respondents' confidence in getting and affording good care (defined as responding "somewhat" or "very" confident to both getting good quality care and affording care). Panel B shows associations with belief that the health system is getting better. Panel C shows associations with perception that the health system needs only minor changes. Poor mental health is defined as respondents reporting "poor" or "fair" mental health on a five-point self-rating scale. Models are unadjusted. Country codes: AR = Argentina, CN = China, CO = Colombia, ET = Ethiopia, GB = United Kingdom, GR = Greece, IN = India, IT = Italy, KE = Kenya, KR = Republic of Korea, LA = Lao PDR, MX = Mexico, NG = Nigeria, PE = Peru, RO = Romania, US = United States, UY = Uruguay, ZA = South Africa. See Appendix Table 4B for exact unadjusted odds ratios and 95% confidence intervals.

**Table E Factors associated with receipt of mental health care among people with poor mental health**

|  | Received care for poor mental health | |
| --- | --- | --- |
|  | Coefficient (OR) | 95% CI |
| Female (ref. male) | 1.51^***^ | [1.28,1.78] |
| Age (ref. <30) |  |  |
| 30-49 | 0.86 | [0.71,1.05] |
| 50+ | 0.69^**^ | [0.55,0.87] |
| Post-secondary education (ref. none, primary or secondary) | 1.19 | [0.99,1.43] |
| Income (ref. low income) |  |  |
| Middle income | 1.09 | [0.90,1.32] |
| High income | 1.15 | [0.93,1.43] |
| Activated patient (ref. not activated) | 0.92 | [0.77,1.11] |
| Urban (ref. rural) | 1.07 | [0.86,1.32] |
| Insurance (ref. none) |  |  |
| Public | 1.36^*^ | [1.00,1.84] |
| Private | 1.36 | [0.97,1.90] |
| Has chronic illness | 2.31^***^ | [1.95,2.74] |
| Usual source (ref. none) |  |  |
| Usual source is poor, fair or good | 1.26^*^ | [1.03,1.54] |
| Usual source is very good or excellent | 1.53^***^ | [1.22,1.92] |
| Country (ref. Ethiopia) |  |  |
| Kenya | 0.88 | [0.37,2.09] |
| Nigeria | 1.22 | [0.51,2.92] |
| South Africa | 3.73^***^ | [2.24,6.19] |
| Peru | 2.29^**^ | [1.39,3.76] |
| Colombia | 1.91^*^ | [1.08,3.36] |
| Mexico | 3.64^***^ | [2.11,6.30] |
| Uruguay | 7.07^***^ | [4.20,11.92] |
| Argentina | 4.78^***^ | [2.83,8.07] |
| Lao PDR | 0.04^***^ | [0.01,0.18] |
| India | 1.59 | [0.93,2.70] |
| China | 0.99 | [0.61,1.59] |
| Rep of Korea | 2.59^***^ | [1.60,4.19] |
| Romania | 2.13^*^ | [1.14,3.98] |
| Greece | 4.03^***^ | [2.39,6.80] |
| Italy | 4.19^***^ | [2.21,7.93] |
| United Kingdom | 6.27^***^ | [3.93,10.00] |
| United States | 7.22^***^ | [4.45,11.72] |
| *N* | 4653 | |

Exponentiated coefficients; 95% confidence intervals in brackets

* p < 0.05, ** p < 0.01, *** p < 0.001

Results from multivariable logistic regression examining factors associated with receipt of mental health care among respondents. Poor mental health is defined as respondents reporting "poor" or "fair" mental health on a five-point self-rating scale. The outcome variable is self-reported receipt of care for mental health in the past 12 months. The model includes gender, age, education, income, patient activation, urban/rural residence, insurance status, chronic illness, quality of usual source of care, and country fixed effects (with Ethiopia as the reference). Odds ratios represent the adjusted likelihood of receiving mental health care for each factor while controlling for all other variables in the model.

**Table F Association between poor mental health status and confidence in the health system, unadjusted**

|  | **Usual source of care** | | **Unmet need** | |
| --- | --- | --- | --- | --- |
| *Poor mental health* | Coefficient (OR) | 95% CI | Coefficient (OR) | 95% CI |
| Ethiopia | 0.88 | [0.69,1.13] | 1.87*** | [1.31,2.65] |
| Kenya | 1.28 | [0.86,1.90] | 1.77* | [1.12,2.78] |
| Nigeria | 0.88 | [0.55,1.42] | 3.29*** | [1.94,5.57] |
| South Africa | 1.33* | [1.02,1.74] | 1.95*** | [1.39,2.74] |
| Peru | 0.86 | [0.63,1.18] | 1.98*** | [1.49,2.63] |
| Colombia | 0.66* | [0.46,0.95] | 3.08*** | [2.18,4.35] |
| Mexico | 1.02 | [0.66,1.57] | 2.26** | [1.34,3.80] |
| Uruguay | 0.84 | [0.43,1.64] | 2.62*** | [1.73,3.96] |
| Argentina | 0.91 | [0.58,1.41] | 2.68*** | [1.87,3.86] |
| Lao PDR | 0.88 | [0.60,1.29] | 0.88 | [0.67,1.17] |
| India | 1.15 | [0.95,1.39] | 1.47 | [0.99,2.17] |
| China | 0.80** | [0.68,0.95] | 1.96*** | [1.32,2.92] |
| Rep. of Korea | 0.80* | [0.65,0.98] | 1.49 | [1.00,2.22] |
| Romania | 1.39 | [0.75,2.57] | 1.63 | [0.94,2.84] |
| Greece | 1.48** | [1.11,1.97] | 2.48*** | [1.69,3.63] |
| Italy | 0.9 | [0.56,1.47] | 1.38 | [0.64,3.00] |
| United Kingdom | 0.8 | [0.57,1.13] | 2.12*** | [1.64,2.75] |
| United States | 0.71 | [0.51,1.00] | 3.89*** | [2.90,5.22] |
|  |  |  |  |  |

Exponentiated coefficients; 95% confidence intervals in brackets

* p < 0.05, ** p < 0.01, *** p < 0.001

P‑values are from two‑sided Wald tests of coefficients in survey‑weighted logistic regression models.

Unadjusted odds ratios (with 95% confidence intervals) from 18 country-specific regression models showing the association between poor mental health status and usual source of care and unmet need for care.

The regression outcomes include respondents reporting they have a (1) usual source of care and (2) an unmet need for medical care in the past year. Poor mental health is defined as respondents reporting "poor" or "fair" mental health on a five-point self-rating scale

**Table G Checklist for Reporting of Survey Studies (CROSS)**

| **Section/topic** | **Item** | **Item description** | **Reported on page #** |
| --- | --- | --- | --- |
| **Title and abstract** | | |  |
| Title and abstract | 1a | State the word “survey” along with a commonly used term in title or abstract to introduce the study’s design. | p.1 and p.3 (tittle and abstract) |
|  | 1b | Provide an informative summary in the abstract, covering background, objectives, methods, findings/results, interpretation/discussion, and conclusions. | p.3 (summary, all paragraphs) |
| **Introduction** | | |  |
| Background | 2 | Provide a background about the rationale of study, what has been previously done, and why this survey is needed. | p.5 (intro 1- intro 4) |
| Purpose/aim | 3 | Identify specific purposes, aims, goals, or objectives of the study. | p.5 (last paragraph) |
| **Methods** | | |  |
| Study design | 4 | Specify the study design in the methods section with a commonly used term (e.g., cross-sectional or longitudinal). | p.6 (Study population) |
|  | 5a | Describe the questionnaire (e.g., number of sections, number of questions, number and names of instruments used). | p.6 (Data source, paragraph 1-2) |
| Data collection methods | 5b | Describe all questionnaire instruments that were used in the survey to measure particular concepts. Report target population, reported validity and reliability information, scoring/classification procedure, and reference links (if any). | p.6-8 (Indicators and reference 17) |
|  | 5c | Provide information on pretesting of the questionnaire, if performed (in the article or in an online supplement). Report the method of pretesting, number of times questionnaire was pre-tested, number and demographics of participants used for pretesting, and the level of similarity of demographics between pre-testing participants and sample population. | p.6 (Data source, paragraph 1-2) |
|  | 5d | Questionnaire if possible, should be fully provided (in the article, or as appendices or as an online supplement). | p.6 (Data source, reference 17) |
| Sample characteristics | 6a | Describe the study population (i.e., background, locations, eligibility criteria for participant inclusion in survey, exclusion criteria). | p.6 (Study population) |
|  | 6b | Describe the sampling techniques used (e.g., single stage or multistage sampling, simple random sampling, stratified sampling, cluster sampling, convenience sampling). Specify the locations of sample participants whenever clustered sampling was applied. | p.6 (Data source, paragraph 1-2) |
|  | 6c | Provide information on sample size, along with details of sample size calculation. | p.6 (Study population) |
|  | 6d | Describe how representative the sample is of the study population (or target population if possible), particularly for population-based surveys. | p. 8 (Statistical analysis, paragraph 1) |
| Survey  administration | 7a | Provide information on modes of questionnaire administration, including the type and number of contacts, the location where the survey was conducted (e.g., outpatient room or by use of online tools, such as SurveyMonkey). | p.6 (Data source) |
|  | 7b | Provide information of survey’s time frame, such as periods of recruitment, exposure, and follow-up days. | p.6 (Data source) |
|  | 7c | Provide information on the entry process:  –>For non-web-based surveys, provide approaches to minimize human error in data entry.  –>For web-based surveys, provide approaches to prevent “multiple participation” of participants. | p.6 (Data source) |
| Study preparation | 8 | Describe any preparation process before conducting the survey (e.g., interviewers’ training process, advertising the survey). | p.6 (Data source) |
| Ethical considerations | 9a | Provide information on ethical approval for the survey if obtained, including informed consent, institutional review board [IRB] approval, Helsinki declaration, and good clinical practice [GCP] declaration (as appropriate). | p.9 (Ethics) |
|  | 9b | Provide information about survey anonymity and confidentiality and describe what mechanisms were used to protect unauthorized access. | p.9 (Ethics) |
| Statistical  analysis | 10a | Describe statistical methods and analytical approach. Report the statistical software that was used for data analysis. | p. 8 (Statistical analysis) |
|  | 10b | Report any modification of variables used in the analysis, along with reference (if available). | p. 7-8 (Mental Health Assessment and Sociodemographic factors) |
|  | 10c | Report details about how missing data was handled. Include rate of missing items, missing data mechanism (i.e., missing completely at random [MCAR], missing at random [MAR] or missing not at random [MNAR]) and methods used to deal with missing data (e.g., multiple imputation). | p. 8 (Statistical analysis) |
|  | 10d | State how non-response error was addressed. | p. 8 (Statistical analysis) |
|  | 10e | For longitudinal surveys, state how loss to follow-up was addressed. | Not applicable |
|  | 10f | Indicate whether any methods such as weighting of items or propensity scores have been used to adjust for non-representativeness of the sample. | p. 8 (Statistical analysis) |
|  | 10g | Describe any sensitivity analysis conducted. | p. 10 (paragraph 4) |
| **Results** | | |  |
| Respondent characteristics | 11a | Report numbers of individuals at each stage of the study. Consider using a flow diagram, if possible. | p. 9 (paragraph 2-3) |
|  | 11b | Provide reasons for non-participation at each stage, if possible. | Appendix Table 1 |
|  | 11c | Report response rate, present the definition of response rate or the formula used to calculate response rate. | Appendix Table 1 |
|  | 11d | Provide information to define how unique visitors are determined. Report number of unique visitors along with relevant proportions (e.g., view proportion, participation proportion, completion proportion). | Not applicable |
| Descriptive  results | 12 | Provide characteristics of study participants, as well as information on potential confounders and assessed outcomes. | p.9 (paragraph 2-3), appendix tables 2-3 |
| Main findings | 13a | Give unadjusted estimates and, if applicable, confounder-adjusted estimates along with 95% confidence intervals and p-values. | p. 10 (paragraph 4), Figures 6-7, Appendix Tables 4-6 |
|  | 13b | For multivariable analysis, provide information on the model building process, model fit statistics, and model assumptions (as appropriate). | p. 8 (Statistical analysis) |
|  | 13c | Provide details about any sensitivity analysis performed. If there are considerable amount of missing data, report sensitivity analyses comparing the results of complete cases with that of the imputed dataset (if possible). | p. 10 (paragraph 4) |
| **Discussion** | | |  |
| Limitations | 14 | Discuss the limitations of the study, considering sources of potential biases and imprecisions, such as non-representativeness of sample, study design, important uncontrolled confounders. | p. 13 (paragraph 4) |
| Interpretations | 15 | Give a cautious overall interpretation of results, based on potential biases and imprecisions and suggest areas for future research. | p. 11-12 (Discussion, paragraphs 1-5) |
| Generalizability | 16 | Discuss the external validity of the results. | p. 12-13 (Discussion, paragraphs 6-10) and p.14-15 |
| **Other sections** | | |  |
| Role of funding source | 17 | State whether any funding organization has had any roles in the survey’s design, implementation, and analysis. | p.4 (Funding) |
| Conflict of interest | 18 | Declare any potential conflict of interest. | Completing interest declaration |
| Acknowledgements | 19 | Provide names of organizations/persons that are acknowledged along with their contribution to the research. | p.15 (Acknowledgments) |
